# Concentration-function coupled electrolytes harmonize thermodynamics and kinetics for stable zinc metal batteries

**DOI:** 10.1039/d5sc05421d

**Published:** 2025-08-27

**Authors:** Tao Liu, Xusheng Dong, Jiashuo Zhang, Huihui Chen, Rongrong Cao, Zixu Sun, Wanhai Zhou, Hongpeng Li, Dongliang Chao, Zhen Zhou, Ruizheng Zhao

**Affiliations:** a Interdisciplinary Research Center for Sustainable Energy Science and Engineering (IRC4SE^2^), Engineering Research Center of Advanced Functional Material Manufacturing of Ministry of Education, School of Chemical Engineering, Zhengzhou University Zhengzhou 450001 Henan China rzzhao@zzu.edu.cn zhenzhou@zzu.edu.cn; b School of Nanoscience and Materials Engineering, Henan University Kaifeng 475004 Henan China; c State Key Laboratory of Molecular Engineering of Polymers and School of Chemistry and Materials, Fudan University Shanghai 200433 China; d College of Mechanical Engineering, Yangzhou University Yangzhou 225127 Jiangsu China; e Key Laboratory of Advanced Energy Materials Chemistry (Ministry of Education), Nankai University Tianjin 300071 China

## Abstract

The application of zinc-based aqueous batteries (ZABs) is limited by poor thermodynamic stability and sluggish electrochemical kinetics due to the unfavorable bulk phase and interface. Conventional electrolyte strategies struggle to balance these aspects. Here, we present a concentration-function coupled electrolyte strategy that enables the independent yet synergistic regulation of bulk and interfacial behaviors of Zn^2+^. Variations in molecular dipole moment, polarity, and concentration determine their coordination with Zn^2+^ and interfacial affinity, enabling the regulation of bulk and interfacial structures, thereby achieving a delicate trade-off between thermodynamics and kinetics. The high-concentration bulk-phase regulator reconstructs the hydrogen-bond network and Zn^2+^ coordination, effectively suppressing hydrogen evolution and zinc corrosion. Meanwhile, the low-concentration interfacial regulator modulates the electric double layer, promoting uniform Zn deposition *via* favorable interfacial chemistry. This electrolyte strategy achieves ultra-stable zinc anodes at room/low temperatures. The strategy's practicality is validated in Zn‖Zn_*x*_V_2_O_5_·*n*H_2_O full cells (95% capacity retention after 4600 cycles), establishing a new paradigm for electrolyte design and offering key insights into the development of durable high-performance ZABs.

## Abstract 2

A concentration–function coupled electrolyte regulates zinc anode thermodynamics and kinetics. With distinct gradients, the dual components exert independent yet synergistic effects, significantly enhancing zinc anode stability.

## Graphical abstract

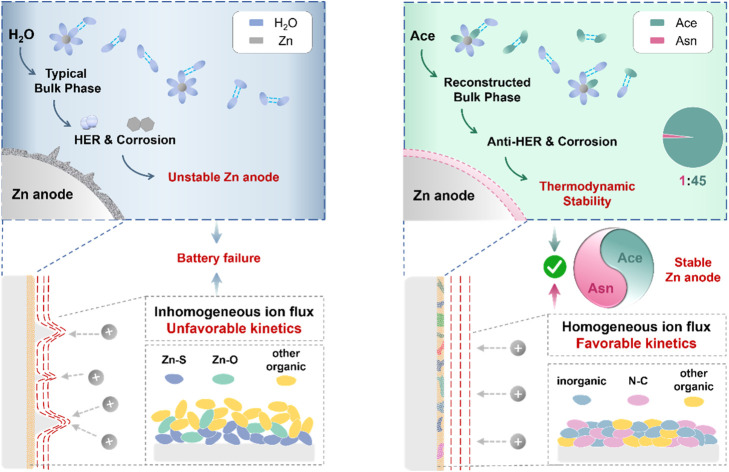

## Introduction

Zinc-based aqueous batteries (ZABs) have emerged as promising candidates for grid-scale energy storage owing to their intrinsic safety, environmental friendliness, and the exceptional properties of metallic zinc anodes. These advantages include high theoretical capacity (820 mAh g^−1^), low cost (approximately 25% of that of lithium), a moderate redox potential (−0.76 V *vs.* the standard hydrogen electrode), and resistance to humid and oxygen-rich conditions.^1–4^ Nevertheless, their practical deployment is impeded by intrinsic issues: thermodynamic instability that triggers severe parasitic reactions (*e.g.*, hydrogen evolution, passivation, and corrosion), and kinetic limitations, which lead to dendrite formation and the accumulation of inactive zinc. These challenges become even more pronounced in extreme environments. Fundamentally, the issues stem from the inability of conventional electrolytes to simultaneously stabilize zinc anode thermodynamics and optimize interfacial kinetics, underscoring the urgent need for novel electrolyte formulations.^5–7^

To resolve this dilemma, researchers have made great efforts in interfacial optimization and electrolyte engineering.^8–10^ Existing strategies generally fall into two categories: interfacial regulators that promote kinetics, and bulk-phase regulators that enhance the thermodynamic stability of zinc anodes. For instance, trace amounts of interfacial regulators such as β-cyclodextrins^11^ or cations^12^ can precisely modulate the electric double layer (EDL) and interfacial chemistry, facilitating Zn^2+^ deposition kinetics and suppressing dendrite growth. However, their low concentrations and limited spatial influence are insufficient to reconstruct the Zn^2+^ solvation environment and inhibit parasitic reactions fundamentally. Conversely, the incorporation of high concentration bulk-phase regulators such as acetamide (Ace)^13^ or acetone^14^ can disrupt the bulk phase through competitive coordination, thereby suppressing water activity and side reactions. Yet, these strong Zn^2+^ solvation and intensified intermolecular interactions deteriorate conductivity and interfacial kinetics thus destabilizing interfacial deposition behavior.^15–17^ This inherent contradiction highlights a fundamental limitation in current single-pathway designs. Most strategies oscillate between thermodynamic stabilization and kinetic enhancement, without achieving simultaneous optimization. Furthermore, few studies have systematically explored the concept of spatially coupled functional roles of electrolyte components to break this conflict and achieve a balance between thermodynamics and kinetics. Such a critical gap in mechanistic understanding and electrolyte design principles restricts the advancement of durable and high-performance ZABs.

Herein, we propose a concentration-function coupled electrolyte (CCE) strategy to address the above contradiction. The dual components with distinct concentrations and functionalities enable independent yet synergistic regulation of bulk and interfacial behaviors. Specifically, a bulk-phase regulator reconstructs the hydrogen-bond (HB) network and solvation structure, effectively suppressing parasitic reactions but at the expense of kinetics, while, the interfacial regulator modulates EDL and interfacial chemistry, guiding uniform Zn^2+^ flux and deposition. This spatially coordinated design achieves a balance between thermodynamic stability and interfacial kinetics performance of the Zn anode, achieving long-term cycling stability under both room and low temperature conditions. The corresponding full cell Zn‖ZVO (Zn_*x*_V_2_O_5_·*n*H_2_O) delivers exceptional rate performance and cycling stability (4600 cycles with 95% capacity retention). This work demonstrates a coupled regulation strategy of bulk and interfacial environments, providing a new paradigm for the rational design of efficient and durable ZABs.

## Results and discussion

### Design principles for the CCE

**Fig. 1:**
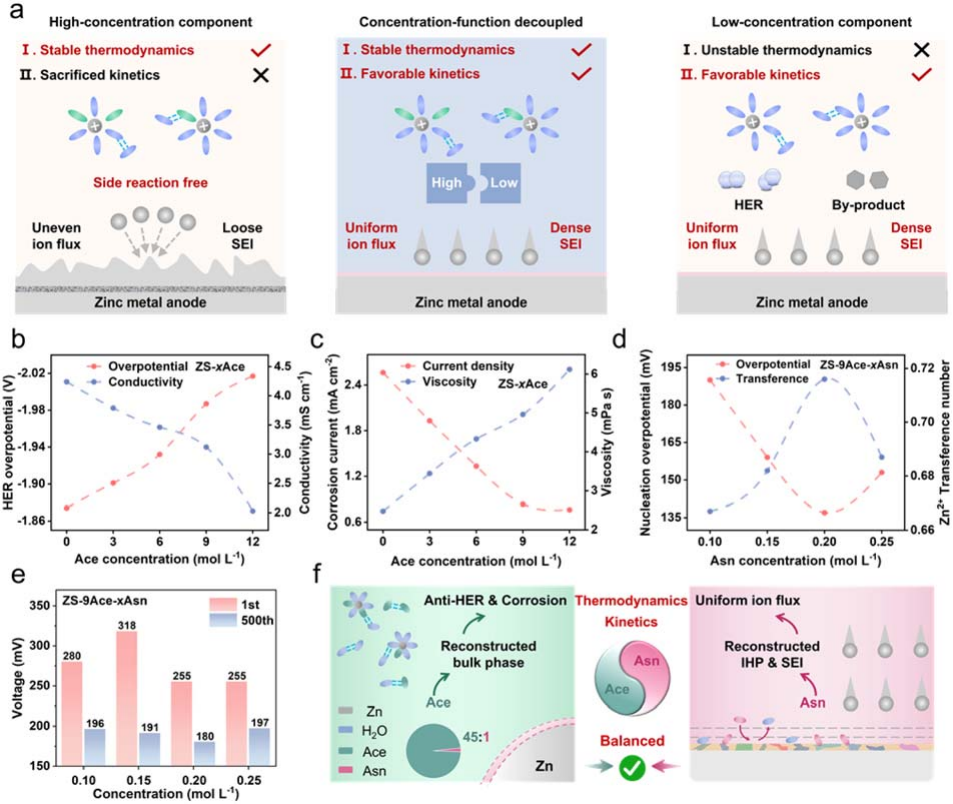
Design framework of the electrolyte and its effect. (a) Schematic illustration of the concentration function coupled strategy. Relationship between (b) hydrogen evolution overpotential and ionic conductivity as well as (c) corrosion current density and viscosity of ZS-*x*Ace electrolytes. (d) Relationship between nucleation overpotential and Zn^2+^ transference number of different electrolytes. (e) Polarization voltage of Zn‖Cu asymmetric cells in ZS-9Ace-*x*Asn at 10 mA cm^−2^. (f) Schematics of the proposed optimal Zn anode with balanced thermodynamics and kinetics enabled by ZS-Dual.

The strong solvation interactions of H_2_O and Zn^2+^ in conventional ZnSO_4_ (ZS) electrolytes induce parasitic reactions. To address this, we employed a concentration-function coupled strategy (Fig. 1a). As a polar and high concentration bulk-phase regulator, Ace was introduced to reconstruct the bulk electrolyte and enhance the thermodynamic stability of Zn anodes. Considering the kinetic sacrifice induced by Ace, we evaluated the hydrogen evolution reaction (HER) overpotential using a three-electrode system and assessed the conductivity *via* SS‖SS tests to identify the optimal Ace concentration (denoted as ZS-*x*Ace, *x* = 3, 6, 9, 12 M, Fig. S1 and S2). As the concentration of the Ace increased, the HER overpotential shifted negatively, while the conductivity gradually decreased (Fig. 1b). Notably, when the concentration increased from 9 M to 12 M, the HER overpotential remained the same, whereas the conductivity dropped sharply. Furthermore, the relationship between corrosion resistance and electrolyte viscosity was systematically investigated (Fig. 1c and S3). As the concentration of Ace increased from 3 M to 9 M, both corrosion current density and viscosity exhibited nearly constant variation trends. However, at ZS-12Ace, the viscosity increased sharply, while the improvement in corrosion resistance was marginal. This is because excessive Ace introduces stronger and more complex interactions, increasing the friction between liquid layers and thereby affecting Zn^2+^ transport and actual electrochemical performance.^18,19^ Therefore, 9 M was determined to be the optimal concentration (denoted as ZS-9Ace), at which the bulk phase is sufficiently reconstructed to suppress side reactions, while the viscosity remains below the threshold that would severely impede ion migration. In addition, it was noticed that the freezing resistance of the electrolyte was further improved when the Ace concentration reached 9 M (Fig. S4 and S5).

However, the high concentration of Ace enhances cationic solvation and intermolecular interactions in the bulk electrolyte, leading to reduced ionic conductivity and interfacial kinetics. To address this, l-Asparagine (Asn) was selected as an interfacial regulator to compensate for sacrificed kinetics, which significantly improved the conductivity (denoted as ZS-*x*Asn, *x* = 0.10, 0.15, 0.20, 0.25 M, Fig. S6). Based on this, we further incorporated Asn into ZS-9Ace to prepare ZS-9Ace-*x*Asn electrolytes (*x* = 0.10, 0.15, 0.20, 0.25 M). To identify the optimal concentration, we measured the nucleation overpotential and the Zn^2+^ transference number in Zn‖Zn symmetric cells (Fig. S7 and S8). Among the tested formulations, ZS-9Ace-0.2Asn exhibited the lowest nucleation overpotential and the highest transference number, effectively mitigating the kinetic limitations (Fig. 1d). Further evaluation of polarization voltage and coulombic efficiency (CE) in Zn‖Cu cells revealed that deviations from the 0.2 M Asn concentration led to increased voltage hysteresis and deteriorated plating/stripping behaviour (Fig. 1e and S9). The accuracy of this optimization was confirmed by the extended cycling stability of Zn‖Zn symmetric cells using the ZS-9Ace-0.2Asn electrolyte, hereafter referred to as ZS-Dual (Fig. S10). It is expected that Ace will reconfigure the bulk phase to enhance the thermodynamic stability of the zinc anode, and Asn will act at the electrode interface to compensate for the sacrificed kinetics caused by Ace (the exact mechanisms will be discussed subsequently). In summary, the zinc anode has a thermodynamic and kinetic balanced relationship (Fig. 1f).

### Mechanistic insights into balanced thermodynamics–kinetics

To gain insight into the CCE strategy and to correlate the structure with electrolyte properties, a series of calculation and experimental characterization studies were performed. The intermolecular interactions in ZS-Dual were analyzed using Fourier transform infrared (FTIR), Raman and ^1^H nuclear magnetic resonance (^1^H NMR) spectroscopy. FTIR was used to examine the structural changes in the electrolytes, and revealed that Ace, rather than Asn, is the main influence on the reconstruction of the intermolecular interactions (Fig. S11 and S12). Specifically, the shift of the sulfate peak at 1100 cm^−1^ reveals a change in the interaction between cations and anions. In the 1550–1700 cm^−1^ region, ZS and ZS-Asn only exhibited simple O–H bending vibrations. However, with the addition of Ace, the intensity and position of the overlapping C=O and N–H stretching peaks changed and shifted significantly, reflecting the strong interactions between C=O and Zn^2+^ as well as H_2_O molecules (Fig. S13). The O–H stretching vibration peak between 3000–3700 cm^−1^ also underwent a significant blueshift and reduced intensity, indicating disruption of the HB network and weakening of the HB between water molecules. This conclusion can be further proved by Raman and ^1^H NMR spectroscopy. The degree of HB disruption was quantified by fitting the O–H stretching band in the Raman spectrum (3000–3700 cm^−1^) to two Gaussian peaks: *I*_w_ (due to HB) and *I*_s_ (due to non-HB).^20,21^ The *I*_w_/*I*_s_ ratio, which reflects HB breakage, followed the order: ZS (0.82) > ZS-Asn (0.73) > ZS-Dual (0.3) > ZS-Ace (0.29), consistent with the thermodynamic stability trend (Fig. S14). Similarly, ^1^H NMR results showed increased chemical shifts for ZS-Ace and ZS-Dual compared with ZS (Fig. S15), suggesting interaction between Ace and the hydroxyl groups of water molecules. This interaction reduces the electron density of H_2_O, leading to higher ^1^H chemical shifts and peaks shifting into the lower field.

**Fig. 2:**
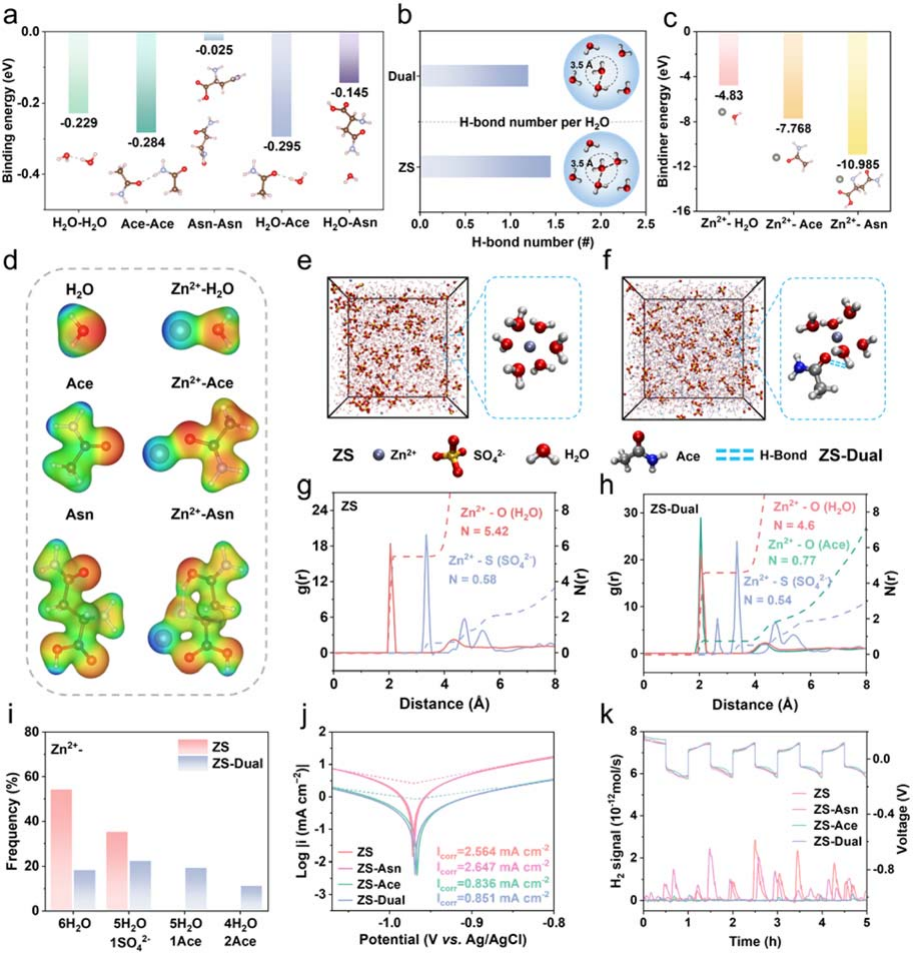
Electrolyte bulk structure and thermodynamic stability of zinc anodes. (a) Sketch of the elementary structure and binding energy of H_2_O, Ace and Asn. (b) The calculated average HB numbers in different electrolytes. (c) The binding energy of H_2_O, Ace and Asn with Zn^2+^. (d) Electrostatic potential mapping. 3D snapshots of the MD simulation for (e) ZS and (f) ZS-Dual electrolytes and schematic of the solvation sheath of Zn^2+^. Cationic radial distribution function g(r) and coordination number *n*(*r*) of different ligands in (g) ZS and (h) ZS-Dual electrolytes. (i) Distribution of the different solvation structures. (j) Tafel plots of the Zn plate tested in different electrolytes. (k) *In situ* DEMS of Zn‖Zn symmetric cells in different electrolytes.

To further explore the causes of the above phenomena, density functional theory (DFT) computations and molecular dynamics (MD) simulations were conducted. The C=O group in Ace serves as a HB acceptor, resulting in a higher binding energy (−0.284 eV) for Ace–H_2_O complexes. This interaction promotes the reorganization of the HB network, thereby stabilizing water molecules in the electrolyte (Fig. 2a). HBs in the bulk phase were quantified according to established HB criteria.^22^ The results indicated that the total number of HBs per water molecule increased from 1.78 to 2.02, while the intermolecular HBs between water molecules decreased from 1.45 to 1.20, demonstrating the formation of a more complex HB network in the presence of Ace (Fig. 2b and S16).

For cation solvation structures, the binding energies of Zn^2+^-Ace (−7.768 eV) and Zn^2+^-Asn (−10.985 eV) are higher than that of Zn^2+^–H_2_O (−4.83 eV), owing to the high polarity and oxygen atom density of their C=O groups (Fig. 2c and d). This indicates that both Ace and Asn have the potential to coordinate with Zn^2+^.^23^ However, spectroscopic analysis reveals that only Ace actually participates in reconstructing the solvation sheath. Despite its relatively weaker binding energy compared with Asn, the higher concentration of Ace enables it to effectively compete with H_2_O, forming Zn^2+^–O=C coordination bonds and thereby reconfiguring the solvation environment. In addition, the larger molecular size of Asn imposes steric hindrance, limiting its accessibility to the solvation sheath. Consequently, Asn does not directly influence the Zn^2+^ desolvation process. MD simulations were performed to further reveal the solvation structure of the electrolyte. As shown in Fig. 2e and f, the concentration dominance of Ace allows it to easily replace water molecules in the Zn^2+^ solvation sheath, thereby modulating the cation solvation structure. The radial distribution function (RDF, Fig. 2g and h) reveals the coordination peaks between Zn^2+^ and the O atoms of Ace as well as H_2_O at 2.0 Å in the ZS-Dual electrolyte, with average coordination numbers (ACN) of 0.77 and 4.6 respectively, indicating that Ace participates in the Zn^2+^ primary solvation sheath. In contrast, in the ZS electrolyte, the ACN of Zn^2+^ with H_2_O is 5.42, significantly higher than the 4.6 observed in the ZS-Dual. This demonstrates that Ace effectively competes with H_2_O, displacing some water molecules in the primary solvation sheath, which is critical for suppressing the HER by reducing water activity. Further analysis of the Zn^2+^ solvation configurations in different electrolytes (Fig. 2i) shows that in ZS, the Zn^2+^–6H_2_O configuration dominates (54%), with Zn^2+^–5H_2_O–SO_4_^2−^ in the secondary solvation sheath (35%). In ZS-Dual, the ratio of these configurations decreases to 18% and 22%, respectively. Notably, the primary solvation sheath in ZS-Dual also contained 19% Zn–5H_2_O-1Ace and 11% Zn–4H_2_O-2Ace, indicating that Ace has replaced part of H_2_O through competitive coordination. This finding is consistent with the previous spectroscopic results. The reduction of solvated water under the action of Ace is conducive to the effective suppression of various side reactions.

Accordingly, the Ace-rich bulk phase structure effectively reduces water activity, inhibits parasitic reactions and enhances the thermodynamic stability of the zinc anode. To verify the enhanced thermodynamic stability, the corrosion behavior of Zn anodes in different electrolytes was first investigated. As shown in Fig. 2j, Tafel fitting reveals a significant reduction in corrosion currents for ZS-Ace (0.836 mA cm^−2^) and ZS-Dual (0.851 mA cm^−2^) compared to ZS (3.769 mA cm^−2^) and ZS-Asn (3.951 mA cm^−2^), suggesting that the restructured bulk phase structure inhibits electrochemical corrosion. After 7 days of immersion, the Zn anodes in ZS-Ace and ZS-Dual remained smooth, while those in ZS showed a hexagonal corrosion product (Zn_4_SO_4_(OH)_6_·5H_2_O) and ZS-Asn presented increased localized pitting corrosion, as confirmed by X-ray diffraction (XRD) (Fig. S17 and S18).^24,25^ It can be hypothesized that the hydrolysis of carboxylate groups in Asn alters the redox potential of Zn, inhibiting the accumulation of alkaline by-products and self-corrosion.^26^ This is further supported by the lower pH in ZS-Asn and ZS-Dual (Fig. S19). To assess the HER, the LSV curves show a negative shift in the reduction potential for Ace (Fig. S20), while a positive shift occurs for Asn, probably due to the lower pH, which promotes the HER. Differential electrochemical mass spectrometry (DEMS) was employed to monitor H_2_ production, revealing substantial H_2_ evolution in ZS and ZS-Asn electrolytes during zinc plating/stripping, whereas the H_2_ signal is weaker in ZS-Ace and ZS-Dual, consistent with the previous analysis (Fig. 2k). The simulation and characterization results highlight the effectiveness of the high-concentration Ace component in stabilizing the thermodynamics of the zinc anode through modulation of the electrolyte bulk phase. However, the complex interactions in the bulk electrolyte with Ace hinder the ordered electrochemical reduction of Zn^2+^. Therefore, Asn must significantly enhance the Zn^2+^ deposition kinetics to achieve a balance between thermodynamic stability and kinetic performance.

**Fig. 3:**
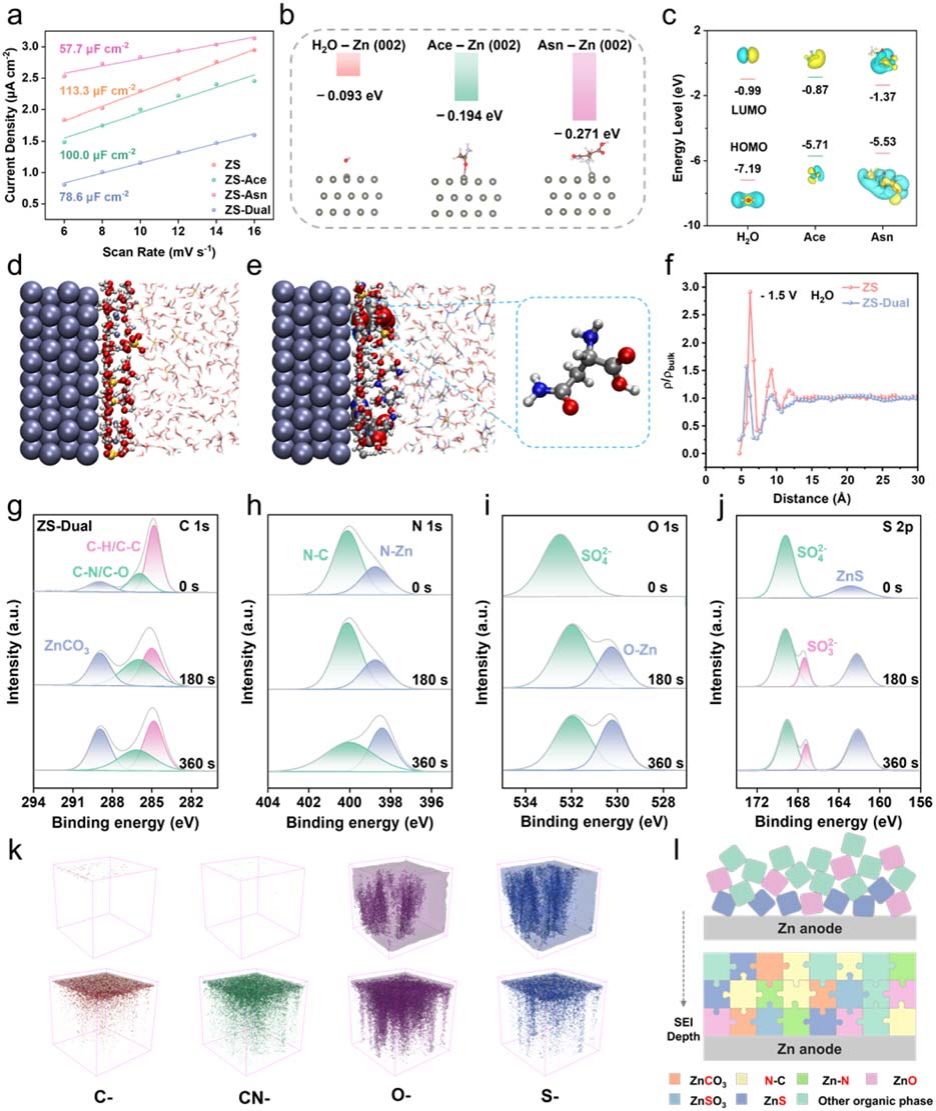
Electric double layer structure and SEI chemistry. (a) The EDLC for Zn substrates in different electrolytes. (b) Comparison of the adsorption energy of different molecules on the Zn (002) slab. (c) DFT calculations on the HOMO and LUMO level of H_2_O, Ace and Asn molecules. (d and e) The snapshots of the EDL structure for the Zn metal interface by MD simulations. (f) Normalized density profiles of H_2_O for the Zn metal surfaces. XPS spectra with in-depth profiles of the Zn anode after 50 cycles with different Ar^+^ sputtering times (0, 180 and 360 s) in ZS-Dual: (g) C 1s, (h) N 1s, (i) O 1s and (j) S 2p spectra. (k) Three-dimensional spatial distribution of elements and compounds of the Zn anode after 50 cycles. (l) Scheme of the SEI on Zn anodes.

The rapid electrodeposition kinetics of Zn^2+^ is largely governed by the properties of the EDL and solid electrolyte interphase (SEI), since these are the main scenarios in which the process occurs. To account for the kinetic factors and gain insight into the electrochemical interface, a series of experiments and simulations were conducted. Electronic double-layer capacitance (EDLC) and differential capacitance curves were used to assess molecular adsorption at the electrode interface.^27,28^ The EDLC values follow the order: ZS-Asn (≈57.7 μF cm^−2^) < ZS-Dual (≈78.6 μF cm^−2^) < ZS-Ace (≈100.0 μF cm^−2^) < ZS (≈113.3 μF cm^−2^), which is attributed to the reduced active area after Asn adsorption and a thicker EDL (Fig. 3a and S21). This trend arises from the preferential localization of Asn in the inner Helmholtz plane (IHP) *via* the amino-Zn coordination, which increases the EDL thickness due to its steric structure.^29^ Differential capacitance curves further support this conclusion (Fig. S22). In addition, Asn exhibited adsorption advantages on different Zn crystal planes (Fig. 3b and S23). This means that they can compete with water molecules and preferentially adsorb on the zinc surface, thereby effectively reducing the tendency of hydrogen evolution reactions at surface active sites. The preferential adsorption of Asn drives the formation of a water-poor EDL at the interface, thereby inhibiting water-induced side reactions. This mechanism is consistent with the results of electrochemical tests. Moreover, Asn exhibits the lowest unoccupied molecular orbital (LUMO) energy level and the narrowest bandgap, suggesting a more favourable electron exchange with the Zn metal (Fig. 3c). MD simulations visualized the EDL structures of ZS and ZS-Dual electrolytes (Fig. 3d, e and S24). In ZS, H_2_O was predominantly adsorbed on the Zn surface, which promotes the possibility of side reactions. In contrast, in ZS-Dual, organic components were competitively adsorbed at the interface, displacing H_2_O. The normalized density profiles of H_2_O molecules at the interface were counted (Fig. 3f), indicating the formation of a water-poor interface. These results confirm the strong IHP adsorption of Asn and its potential to modulate interfacial chemistry.^30^

To investigate the interfacial chemistry under the CCE strategy, the Zn anode after 50 cycles in different electrolytes was analysed by X-ray photoelectron spectroscopy (XPS) and time-of-flight secondary ion mass spectrometry (ToF-SIMS). As shown in Fig. 3g–j and S2, the C 1 s spectrum of ZS-Dual reveals an increased ZnCO_3_ signal at 288.9 eV, probably due to the sufficient reduction of Ace and Asn. Enhanced C–C/C–H (284.8 eV) and C–O/C–N (285.9 eV) peaks also suggest significant organic accumulation at the Zn interface (Fig. 3g and S25a, e, i).The N 1s spectrum exhibits two features: a dominant peak at 400.0 eV corresponding to the amide groups (–CONH_2_) of absorbed Asn, and a minor peak at 398.4 eV attributed to –NH_3_^+^ interactions with the Zn surface, implying the physisorption of Asn at the IHP (Fig. 3h). In the O 1 s region, ZS exhibits a dominant peak at 531.5 eV (Fig. S25c), which in combination with XRD and SEM results, can be attributed to zinc hydroxide sulfate (ZHS) by-products (Fig. S17 and S18). In contrast, ZS-Ace shows no obvious by-products in XRD/SEM and no O–Zn peak in XPS, confirming the suppression of side reactions (Fig. S25g). Notably, ZS-Asn and ZS-Dual exhibit O–Zn peaks at ∼530.0 eV, combined with the absence of ZHS, which revealed that Asn-induced inorganic SEI components have formed rather than ZHS (Fig. 3i and S25k). ZnO, known for its mechanical strength, can inhibit dendrite growth and act as an electrolyte barrier.^12,31^ The S 2p spectrum reveals stratified sulphur species, with SO_3_^2−^ dominating the SEI subsurface (Fig. 3j). This is attributed to preferential SO_4_^2−^ reduction, driven by the distinctive EDL structure formed through the concentration-function coupled strategy.

The ToF-SIMS depth profile reveals the spatial distribution of elements within the SEI (Fig. 3k). In ZS-Dual, stable secondary ion signals for C–, CN–, O–, and S– indicate the formation of a robust and uniform interfacial layer. In contrast, ZS exhibits rapid signal decay, suggesting an unstable interphase formation caused by severe parasitic reactions. Additionally, the Zn (inorganic) signal in ZS-Dual is significantly stronger than that in ZS (Fig. S26), further confirming the formation of an inorganic-rich SEI, consistent with the XPS results (Fig. 3l). Although both amide molecules possess similar functional groups, they exhibit distinct functional behaviours in the system. Beyond concentration differences, the additional amino side chain in Asn introduces a more complex molecular structure, resulting in a shift in the positive/negative charge centres leading to a change in the dipole moment, which are also factors contributing to this phenomenon. Furthermore, the amino group, acting as an electron-donating group, strongly interacts with the Zn surface through coordination bonding. This adsorption not only anchors the molecule but also induces the formation of an SEI, thereby facilitating local electronic redistribution on Zn and uniform nucleation.

At low temperature, the SEI composition differs from that at room temperature (Fig. S27). Formation of inorganic motifs (N–Zn and O–Zn) is suppressed, and component ratios shift; for ZS-Dual, ZnCO_3_ and SO_3_^2−^, signals are weaker than those at room temperature, indicating restrained SEI growth. Nevertheless, Zn anodes in CCE electrolytes form a conformal, uniform SEI under both conditions, retaining an interface favourable for Zn deposition kinetics.

**Fig. 4:**
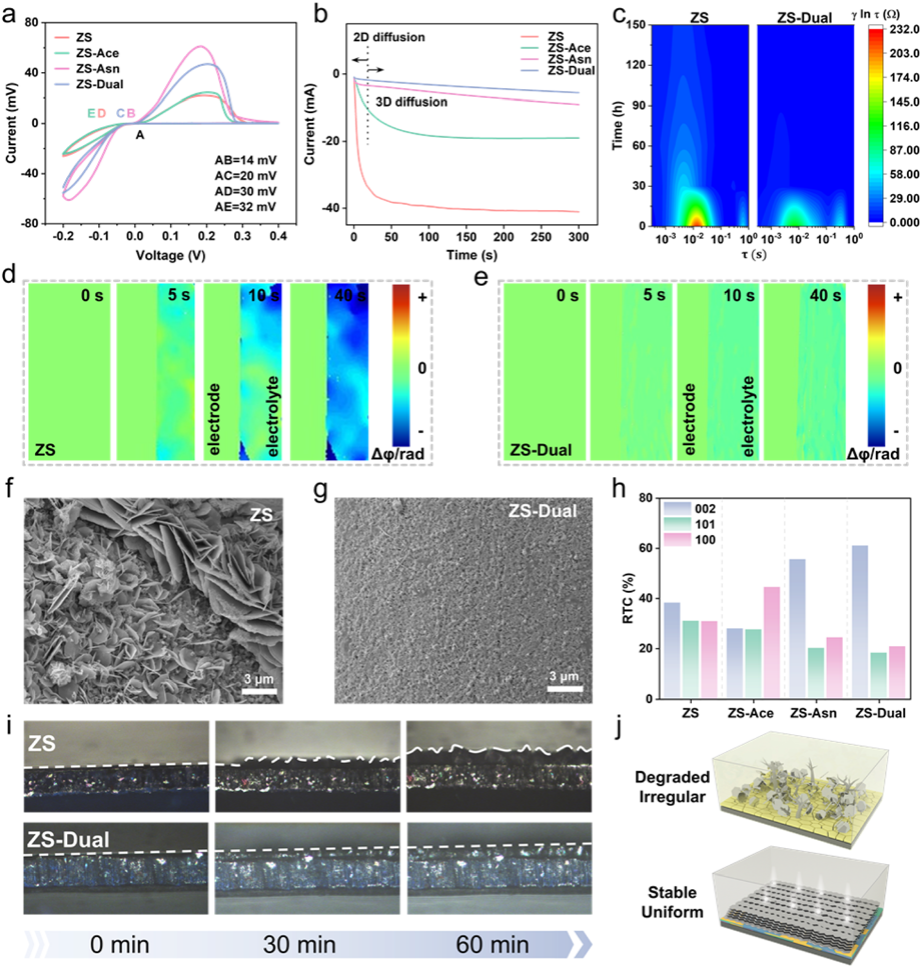
Kinetics and structural evolution of zinc anodes. (a) CV curves for Zn nucleation of the Zn‖Cu asymmetric cell in different electrolytes. (b) CA curves at an overpotential of −150 mV. (c) *In situ* DRT spectra of Zn‖Zn symmetric cells in different electrolytes. *In situ* EDH of the evolution of the Zn^2+^ concentration at the electrode interface in (d) ZS and (e) ZS-Dual electrolytes. Planar SEM of Zn deposits with 1 mAh cm^−2^ in (f) ZS and (g) ZS-Dual electrolytes. (h) RTC of Zn deposits in a Zn‖Zn symmetric cell. (i) *In situ* optical microscopy images of Zn plating at 10 mA cm^−2^. (j) Schematics of the interfacial behavior of Zn anodes cycled in ZS and ZS-Dual electrolytes.

The Asn-regulated IHP and the inorganically enriched SEI play a critical role in enhancing interfacial kinetics. The H_2_O-depleted IHP suppresses parasitic reactions, while the high surface energy of the SEI promotes vertical Zn^2+^ migration, enabling dense Zn single crystal deposition.^30,31^ To quantify these improvements, a series of electrochemical tests were implemented. Cyclic voltammetry (CV) revealed that the nucleation overpotentials in ZS-Asn and ZS-Dual were reduced by 10–20 mV, indicating lower nucleation barriers and improved deposition kinetics (Fig. 4a). The activation energy (*E*_a_) of ZS-Asn and ZS-Dual calculated *via* the Arrhenius equation was significantly lower than that of ZS and ZS-Ace (Fig. S28 and S29). This is because, although Asn does not enter the Zn^2+^ solvation sheath, its regulation of the IHP and SEI effectively reduces the overall electrochemical reaction barrier.^32–34^ Chronoamperometry (CA) further revealed differences in Zn^2+^ diffusion behaviour. As shown in Fig. 4b, ZS-Dual quickly transitioned from a 2D nucleation phase to a 3D diffusion process, promoting orderly vertical migration of Zn^2+^ and uniform deposition. In contrast, the current of ZS exhibited a continuous decrease within 300 s, indicating an uncontrolled 2D Zn^2+^ diffusion, which induces initial protrusion and triggers the ‘tip effect’. Notably, the ZS-Ace showed deteriorating kinetics in all of the above tests. This is due to the fact that high concentrations of the bulk-phase regulator significantly enhance intermolecular forces in solution, resulting in a sacrifice in kinetics (consistent with our pre-screening behaviour).

*In situ* electrochemical impedance spectroscopy (EIS) was performed on Zn‖Zn symmetric cells using ZS and ZS-Dual electrolytes (Fig. S30). The resulting spectra were analyzed *via* distribution of relaxation times (DRT) to assess interfacial kinetics. The relaxation times (*τ*) at ∼0.01 s correspond to the charge transfer resistance *R*_ct_ (Fig. 4c). During the initial plating process, ZS-Dual exhibited a significantly lower *R*_ct_ than ZS, and quickly stabilized, eventually showing no distinct *R*_ct_ signal, indicating enhanced Zn^2+^ transport kinetics and stable deposition. The initially smaller *τ* further reflects faster interfacial reaction kinetics.^35–37^ Typically, a mismatch between slow mass transfer and fast charge/discharge leads to sharp Zn^2+^ concentration gradients at the electrode/electrolyte interface, which promotes dendrite formation.^38^ To visualize this process, *in situ* electrochemical digital holography (EDH) was used. Specifically, during the electrochemical process, the amplitude and phase changes at the electrode/electrolyte interface were recorded, thus revealing the dynamic evolution of the concentration field and the diffusion layer (Fig. 4d, e and S31).^39–41^ In ZS and ZS-Ace, pronounced Zn^2+^ concentration gradients were observed, indicating inhomogeneous ion distribution. In contrast, ZS-Asn and ZS-Dual retained a stable and uniform Zn^2+^ diffusion layer, effectively preventing localized ion accumulation and irreversible crystal formation.

The deposition morphology of Zn was investigated by scanning electron microscopy (SEM). The initial Zn deposition behavior reveals irregular and moss-like deposits in ZS and ZS-Ace, which indicates uncontrolled dendrite growth (Fig. 4f and S32a), while ZS-Dual and ZS-Asn showed a dense and flat morphology (Fig. 4g and S32b). These results suggest that ZS and ZS-Ace lack the ability to guide uniform Zn deposition. XRD analysis was performed to investigate the microstructure of the Zn deposition (Fig. S33). A pronounced enhancement in the Zn (002) plane was observed in the presence of Asn, corresponding to preferred crystal orientation and uniform deposition. Relative texture coefficients (RTCs) further confirmed this trend (Fig. 4h). ZS exhibited a disordered growth pattern, while ZS-Ace favoured the Zn (101) plane due to hindered electrodeposition kinetics, which is unfavourable for uniform deposition. In contrast, ZS-Asn and ZS-Dual showed a strong preference for Zn (002) and decreased Zn (100) and (101) planes, indicating single crystal and oriented growth. The Zn deposition morphology was examined at low temperatures (Fig. S34). ZS-Ace retained a loose, porous structure, whereas ZS-Dual produced a denser, more compact deposit. Cooling slowed Zn deposition and enlarged Zn nuclei, but the overall deposition morphology remained comparable to that at room temperature.

The surface morphology of Zn foil after 50 cycles in Zn‖Zn symmetric cells with different electrolytes was evaluated by SEM (Fig. S35) and atomic force microscopy (AFM, Fig. S36). ZS led to a chaotic dendritic morphology with a lot of cavities due to irregular deposits, by-products, and severe hydrogen evolution. ZS-Ace and ZS-Asn showed non-uniform deposition and corrosion voids, respectively, due to their unbalanced kinetics and thermodynamics. In comparison, ZS-Dual produced smooth, dense Zn layers, as confirmed by both SEM and 3D AFM imaging. *In situ* optical microscopy at 10 mA cm^−2^ further visualized the Zn growth kinetics (Fig. 4i). After 30 min of plating in ZS electrolyte, irregular protrusions appeared on the Zn anode surface. Driven by the “tip effect”, the dendrites grow rapidly and eventually form a thick and dendrite-rich deposition layer after 60 min. In contrast, the Zn anode in ZS-Dual retained a flat, uniform and dense surface throughout the deposition period, despite the deposition of higher capacity Zn. This facilitated Zn deposition behaviour benefits from the modified IHP and the inorganic-rich SEI. Asn adsorbs on the IHP, shielding unfavourable nucleation sites, while the inorganic component in the SEI increases the surface energy of the Zn anode, collectively creating a stable and uniform interface (Fig. 4j).

### Electrochemical performance of ZABs

**Fig. 5:**
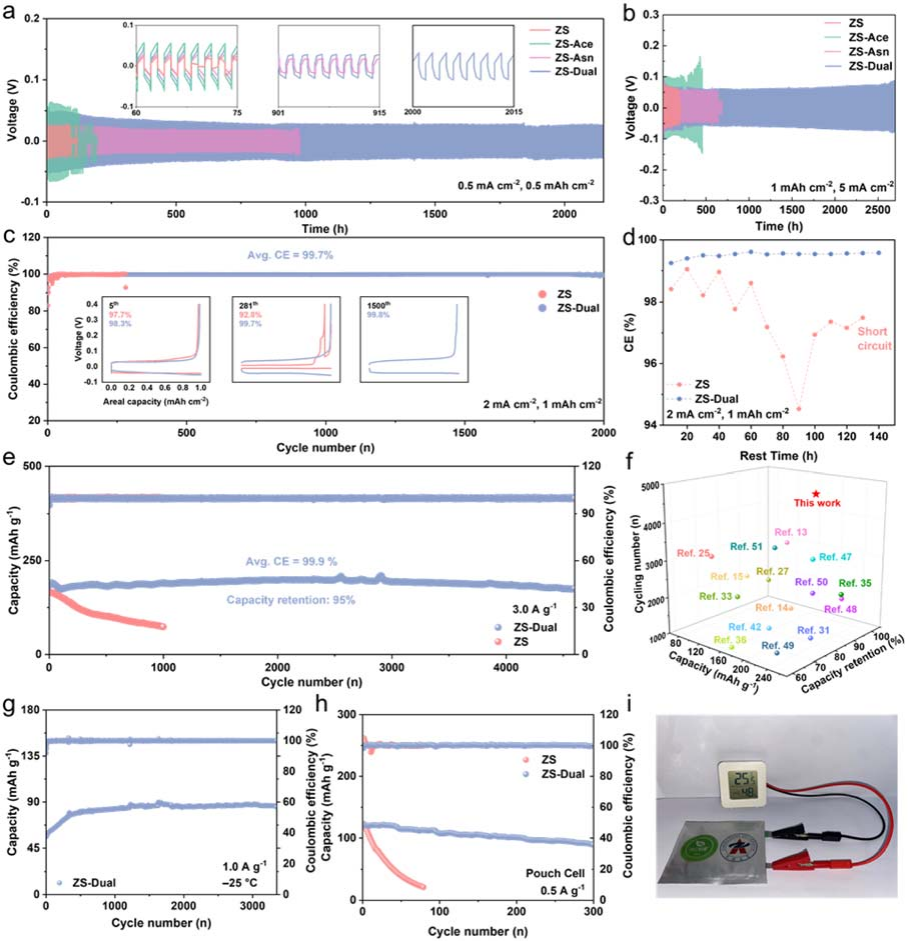
Electrochemical performance. Galvanostatic Zn plating/stripping in a Zn‖Zn symmetric cell at (a) 0.5 mA cm^−2^, 0.5 mAh cm^−2^ and (b) 5 mA cm^−2^, 1 mAh cm^−2^ at 25 °C. (c) CE of Zn plating/stripping in a Zn‖Cu asymmetric cell at 2 mA cm^−2^, 1 mAh cm^−2^. (d) CE of Zn plating/stripping during sustained resting. (e) Cycle performance and corresponding CE of Zn‖ZVO full cells at 25 °C with 3.0 A g^−1^. (f) Cycling performance comparison with recent studies. (g) Cycle performance at −25 °C with 1.0 A g^−1^. (h) Cycle performance of Zn‖ZVO pouch cells with a size of 4 cm × 4 cm at 0.5 A g^−1^ and 25 °C. (i) Demonstration of a single-layer Zn‖ZVO full cell powering a humidity/temperature sensor.

The CCE strategy effectively boosts the reversibility of the Zn anode, as demonstrated in both Zn‖Zn symmetric cells and Zn‖Cu asymmetric cells. As shown in Fig. 5a, the Zn‖Zn cell with the ZS electrolyte fails after only 90 h at 0.5 mA cm^−2^ and 0.5 mAh cm^−2^, due to side reactions and dendrite growth driven by poor thermodynamic and kinetic control. When ZS-Ace or ZS-Asn electrolytes are used individually, the cycle life is extended to 130 h and 970 h, respectively, still limited by the imbalance between thermodynamics and kinetics. In contrast, the ZS-Dual electrolyte achieves a markedly extended lifespan of 2150 h, highlighting the synergistic stabilization and kinetic enhancement provided by the combined additives. The voltage hysteresis increases in the order: ZS-Asn (18 mV) < ZS (24 mV) < ZS-Dual (26 mV) < ZS-Ace (60 mV), underlining the critical role of Asn in improving kinetics. Even at higher current densities and capacities, the ZS-Dual is also capable of achieving over 6700 stable cycles (Fig. 5b). In addition, higher depth of discharge (DOD) can be achieved in the ZS-Dual electrolyte (Fig. S37). The Zn‖Zn cell cycles for more than 240 h at a DOD of 34%, which results from the synergistic effect of the dual component, and avoids the excessive consumption of Zn metal. ZS-Dual also exhibits superior rate performance compared to the ZS electrolyte (Fig. S38). Furthermore, at −25 °C and 0.5 mA cm^−2^, ZS-Dual enables a remarkable cycle life of 2200 h (Fig. S39), whereas ZS-Ace fails prematurely due to its further limited kinetic capability in low temperature environments. In addition, ZS-Dual exhibits a satisfactory lifespan at 1 mA cm^−2^ (Fig. S40).

To further evaluate the reversibility of the Zn anode, long-term coulombic efficiency (CE) tests were conducted on Zn‖Cu asymmetric cells using different electrolytes (Fig. 5c). The Zn‖Cu asymmetric cell in the ZS electrolyte failed after just 281 cycles due to severe dendrite formation and uncontrolled side reactions. In contrast, the Zn‖Cu asymmetric cell in the ZS-Dual electrolyte retained an average CE of 99.7% over 2000 cycles. In addition, the initial CE increased from 83% to 90%, indicating reduced irreversible losses and a more efficient formation process. Further increasing the current density to 10 mA cm^−2^, the Zn‖Cu asymmetric cell in ZS-Dual exhibited an average CE of 99.9% over 2500 cycles (Fig. S41). In weakly acidic systems, calendar ageing may be the main cause of shortened zinc anode life, rather than cyclic ageing, and continuous plating/stripping can obscure parasitic effects to produce higher pseudo-CE.^42^ To isolate these, a 10 h rest period was introduced between each cycle to allow parasitic reactions to fully proceed (Fig. 5d).^43^ Under these conditions, the ZS electrolyte showed sharp CE fluctuations after 40 h and failed completely after 140 h. In contrast, ZS-Dual retained a stable CE above 99.5%, even during 140 h of cumulative rest, highlighting superior interfacial stability. The capacity voltage curves (Fig. S42) further confirmed this trend. ZS exhibited increasing polarization and signs of short-circuiting after 80 h, while ZS-Dual retained highly consistent voltage profiles, underscoring its improved thermodynamic stability and dendrite suppression capabilities.

To further validate the practical applicability of the ZS-Dual electrolyte, full cells were assembled using Zn_*x*_V_2_O_5_·*n*H_2_O (ZVO) as the cathode. The synthesized ZVO exhibited consistent XRD patterns and SEM morphology compared to previous reports (Fig. S43).^44,45^ The CV curves of Zn‖ZVO cells with different electrolytes showed similar redox peaks, indicating that the intrinsic energy storage mechanism of ZVO remained unchanged (Fig. S44). EIS revealed a prominent semicircle in the high-frequency region for the ZS-Dual system, confirming the formation of a stable SEI layer, which was scarcely detectable with the ZS electrolyte (Fig. S45).^11^ Additionally, the reduced semicircle radius indicates lower interfacial polarization. To further assess the interfacial kinetics, DRT analysis was performed (Fig. S46). The ZS-Dual based full cell exhibited significantly lower *R*_ct_ and *R*_diffusion_ values, indicating significantly suppressed polarization and enhanced interfacial kinetics. Additionally, the shortened relaxation time corresponding to *R*_ct_ and *R*_diffusion_ further supports this conclusion. Notably, a peak at *τ* ∼10^−5^ corresponds to *R*_SEI_, consistent with the small semicircle observed in the EIS spectra.

The long-term cycling stability of the Zn‖ZVO cells was evaluated in both ZS and ZS-Dual electrolytes. As shown in Fig. 5e, the Zn‖ZVO cell with the ZS electrolyte retained only 44% of its initial capacity after 1000 cycles at 3 A g^−1^, primarily due to thermodynamic and kinetic deficiencies. In contrast, the cell in the ZS-Dual electrolyte exhibited an initial capacity of 185.2 mAh g^−1^ and showed 95% capacity retention after 4600 cycles, with nearly 100% CE. This result is much better than the previously reported electrolyte strategies (Fig. 5f), reflecting the superiority of the CCE strategy.^46–51^ At −25 °C, the ZS-Dual electrolyte also enabled excellent low temperature cycling stability, with the Zn‖ZVO cell delivering stable performance over 3500 cycles at 0.1 A g^−1^ with no noticeable capacity degradation (Fig. 5g and S47). In comparison, the ZS-Ace electrolyte failed to achieve satisfactory performance under the same conditions (Fig. S48). The gradual increase in capacity during the initial cycles in ZS-Dual is attributed to the slow process of SEI formation, a phenomenon not observed in ZS-Ace. The rate performance of the Zn‖ZVO cells further highlights the advantage of the ZS-Dual system. As shown in Fig. S49 and S50, the cell delivered higher specific capacities across a wide range of current densities (0.5–15 A g^−1^), with the corresponding charge/discharge curves demonstrating superior rate capability outperforming cells with the ZS electrolyte. Furthermore, the self-discharge behaviour of the full cell was also examined. After 48 h of rest, the Zn‖ZVO cell with ZS-Dual retained 88.7% of its initial capacity, while the cell with the ZS electrolyte showed a drastic decrease, retaining only 76.1%, due to pronounced side reactions (Fig. S51). To further evaluate the practicality of the cells, the Zn‖ZVO pouch cells were assembled. ZS-Dual delivered a capacity of 101 mAh g^−1^ (83% of the initial capacity) after 200 cycles, while ZS deteriorated rapidly (Fig. 5h). It is worth noting that the pouch cell with the ZS electrolyte exhibits obvious swelling after cycling (Fig. S52), implying a drastic HER, which is one of the reasons for its rapid failure. As shown in Fig. 5i, the energy provided by a single-layer pouch cell successfully powered a humidity/temperature sensor. These results highlight the significance of the CCE design strategy and its potential for practical application in high safety, and long-life ZABs.

## Conclusions

In conclusion, a CCE design strategy was developed to harmonize the thermodynamic stability and interfacial kinetics of zinc anodes. Comprehensive MD/DFT calculations and experimental characterization revealed the synergistic roles of Ace and Asn in regulating the bulk electrolyte and interfacial chemistry. The high concentration Ace effectively modulates the bulk phase to inhibit side reactions, thereby enhancing thermodynamic stability, albeit with compromised kinetics, whereas the low concentration Asn regulates the inner EDL and SEI, promotes uniform ion flux, and facilitates dendrite-free Zn deposition, thereby restoring interfacial kinetics. Consequently, the Zn‖Zn symmetric cells utilizing this hybrid electrolyte demonstrated stable cycling without hydrogen evolution or dendrite formation for over 2000 h and 6700 cycles (at 1 mA cm^−2^ and 5 mA cm^−2^ respectively) at room temperature and 2150 h under low temperature conditions. Moreover, the Zn‖Cu asymmetric cells show suppressed calendar ageing and impressive CE during intermittent quiescence. The corresponding full cell Zn‖ZVO retains 95% capacity after 4600 cycles. This study expands the electrolyte design paradigm for ZABs and offers a promising strategy for balancing thermodynamic and kinetic requirements.

## Author contributions

R. Z. and Z. Z. supervised the research. T. L. conceived, conducted, collected, and analysed the experimental data as well as wrote the manuscript. X. D. J. Z. H. C. and R. C. contributed to the suggestions of the research and performed the experimental data analysis. Z. S., W. Z., H. L. and D. C. provided theoretical guidance. All authors discussed the results and commented on the manuscript.

## Conflicts of interest

The authors declare no conflicts of interest.

## Supplementary Material

SC-016-D5SC05421D-s001

## Data Availability

The original data supporting this article are available in the main context and SI. See DOI: https://doi.org/10.1039/d5sc05421d.

## References

[cit1] Xu J., Ji X., Zhang J., Yang C., Wang P., Liu S., Ludwig K., Chen F., Kofinas P., Wang C. (2022). Aqueous electrolyte design for super-stable 2.5 V LiMn2O4 ‖ Li4Ti5O12 pouch cells. Nat. Energy.

[cit2] Zhao R., Elzatahry A., Chao D., Zhao D. (2022). Making MXenes more energetic in aqueous battery. Matter.

[cit3] Dai Y., Lu R., Zhang C., Li J., Yuan Y., Mao Y., Ye C., Cai Z., Zhu J., Li J., Yu R., Cui L., Zhao S., An Q., He G., Waterhouse G. I. N., Shearing P. R., Ren Y., Lu J., Amine K., Wang Z., Mai L. (2024). Zn2+-mediated catalysis for fast-charging aqueous Zn-ion batteries. Nat. Catal..

[cit4] Xu J., Liu T., Dong X., Dong X., Zhou W., Li X., Chao D., Zhou Z., Zhao R. (2025). Challenges and opportunities in 2D materials for high-performance aqueous ammonium ion batteries. Natl. Sci. Rev..

[cit5] Liu S., Zhang R., Mao J., Zhao Y., Cai Q., Guo Z. (2022). From room temperature to harsh temperature applications: Fundamentals and perspectives on electrolytes in zinc metal batteries. Sci. Adv..

[cit6] Guo P., Zhao R., Zhang Z., Li J., Zhang W., Wang A., Kang T., Lian C., Guo Z., Wang J., Zhang J., Ma Y. (2024). Droplet-Directed Anisotropic Assembly of Semifootball-Like Carbon Nanoparticles with Multimodal Pore Architectures. Adv. Funct. Mater..

[cit7] Sui D., Luo R., Xie S., Zhang H., Ma T., Sun H., Jia T.-T., Sun J., Li X. (2024). Atomic ruthenium doping in collaboration with oxygen vacancy engineering boosts the hydrogen evolution reaction by optimizing H absorption. Chem. Eng. J..

[cit8] Chen W., Wang Y., Wang F., Zhang Z., Li W., Fang G., Wang F. (2024). Zinc Chemistries of Hybrid Electrolytes in Zinc Metal Batteries: From Solvent Structure to Interfaces. Adv. Mater..

[cit9] Guo X., Zhang S., Hong H., Wang S., Zhu J., Zhi C. (2025). Interface regulation and electrolyte design strategies for zinc anodes in high-performance zinc metal batteries. iScience.

[cit10] Wang M., Xu Z., He C., Cai L., Zheng H., Sun Z., Liu H. K., Ying H., Dou S. (2025). Fundamentals, Advances and Perspectives in Designing Eutectic Electrolytes for Zinc-Ion Secondary Batteries. ACS Nano.

[cit11] Luo J., Xu L., Yang Y., Huang S., Zhou Y., Shao Y., Wang T., Tian J., Guo S., Zhao J., Zhao X., Cheng T., Shao Y., Zhang J. (2024). Stable zinc anode solid electrolyte interphase *via* inner Helmholtz plane engineering. Nat. Commun..

[cit12] Pastel G. R., Pollard T. P., Liu Q., Lavan S., Zhu Q., Jiang R., Ma L., Connell J., Borodin O., Schroeder M. A., Zhang Z., Xu K. (2024). Designing interphases for highly reversible aqueous zinc batteries. Joule.

[cit13] Wang S., Chen S., Ying Y., Li G., Wang H., Cheung K. K. K., Meng Q., Huang H., Ma L., Zapien J. A. (2024). Fast Reaction Kinetics and Commendable Low-Temperature Adaptability of Zinc Batteries Enabled by Aprotic Water-Acetamide Symbiotic Solvation Sheath. Angew. Chem., Int. Ed..

[cit14] Cao X., Xu W., Zheng D., Wang F., Wang Y., Shi X., Lu X. (2024). Weak Solvation Effect Induced Optimal Interfacial Chemistry Enables Highly Durable Zn Anodes for Aqueous Zn-Ion Batteries. Angew. Chem., Int. Ed..

[cit15] Cong J., Wang Y., Lin X., Huang Z., Wang H., Li J., Hu L., Hua H., Huang J., Lin Y. C., Xu H., Li Z., Huang Y. (2025). Kinetics Compensation Mechanism in Cosolvent Electrolyte Strategy for Aqueous Zinc Batteries. J. Am. Chem. Soc..

[cit16] Yu X., Chen M., Li Z., Tan X., Zhang H., Wang J., Tang Y., Xu J., Yin W., Yang Y., Chao D., Wang F., Zou Y., Feng G., Qiao Y., Zhou H., Sun S.-G. (2024). Unlocking Dynamic Solvation Chemistry and Hydrogen Evolution Mechanism in Aqueous Zinc Batteries. J. Am. Chem. Soc..

[cit17] Zhao R., Dong X., Liang P., Li H., Zhang T., Zhou W., Wang B., Yang Z., Wang X., Wang L., Sun Z., Bu F., Zhao Z., Li W., Zhao D., Chao D. (2023). Prioritizing Hetero-Metallic Interfaces *via* Thermodynamics Inertia and Kinetics Zincophilia Metrics for Tough Zn-Based Aqueous Batteries. Adv. Mater..

[cit18] Rodrigues M.-T. F., Babu G., Gullapalli H., Kalaga K., Sayed F. N., Kato K., Joyner J., Ajayan P. M. (2017). A materials perspective on Li-ion batteries at extreme temperatures. Nat. Energy.

[cit19] Yao N., Yu L., Fu Z.-H., Shen X., Hou T.-Z., Liu X., Gao Y.-C., Zhang R., Zhao C.-Z., Chen X., Zhang Q. (2023). Probing the Origin of Viscosity of Liquid Electrolytes for Lithium Batteries. Angew. Chem., Int. Ed..

[cit20] Jiang L., Han S., Hu Y.-C., Yang Y., Lu Y., Lu Y.-C., Zhao J., Chen L., Hu Y.-S. (2024). Rational design of anti-freezing electrolytes for extremely low-temperature aqueous batteries. Nat. Energy.

[cit21] Wang Y.-H., Zheng S., Yang W.-M., Zhou R.-Y., He Q.-F., Radjenovic P., Dong J.-C., Li S., Zheng J., Yang Z.-L., Attard G., Pan F., Tian Z.-Q., Li J.-F. (2021). In situ Raman spectroscopy reveals the structure and dissociation of interfacial water. Nature.

[cit22] Bai X., Chen C., Zhao X., Zhang Y., Zheng Y., Jiao Y. (2024). Accelerating the Reaction Kinetics of CO(2) Reduction to Multi-Carbon Products by Synergistic Effect between Cation and Aprotic Solvent on Copper Electrodes. Angew. Chem., Int.
Ed..

[cit23] Wang D., Lv D., Liu H., Zhang S., Wang C., Wang C., Yang J., Qian Y. (2022). In Situ Formation of Nitrogen-Rich Solid Electrolyte Interphase and Simultaneous Regulating Solvation Structures for Advanced Zn Metal Batteries. Angew. Chem., Int. Ed..

[cit24] Wang X., Zhou W., Wang L., Zhang Y., Li S., Li X., Zhao Z., Zhang T., Jin H., Song X., Liang P., Zhang B., Zhao D., Chao D. (2025). Benchmarking Corrosion with Anionic Polarity Index for Stable and Fast Aqueous Batteries Even in Low-Concentration Electrolyte. Adv. Mater..

[cit25] Liu T., Dong X., Tang B., Zhao R., Xu J., Li H., Gao S., Fang Y., Chao D., Zhou Z. (2024). Engineering electrolyte additives for stable zinc-based aqueous batteries: Insights and prospects. J. Energy Chem..

[cit26] Wang D., Li Q., Zhao Y., Hong H., Li H., Huang Z., Liang G., Yang Q., Zhi C. (2022). Insight on Organic Molecules in Aqueous Zn-Ion Batteries with an Emphasis on the Zn Anode Regulation. Adv. Energy Mater..

[cit27] Wu Q., Zhang J., Yang S., Luo F., Yan Z., Liu X., Xie H., Huang J., Chen Y. (2025). Bridging Electrolyte Bulk and Interfacial Chemistry: Dynamic Protective Strategy Enable Ultra-Long Lifespan Aqueous Zinc Batteries. Angew. Chem., Int. Ed..

[cit28] Herzog G., Moujahid W., Strutwolf J., Arrigan D. W. (2009). Interactions of proteins with small ionised molecules: electrochemical adsorption and facilitated ion transfer voltammetry of haemoglobin at the liquid/liquid interface. Analyst.

[cit29] Qin S., Zhang J., Xu M., Xu P., Zou J., Li J., Luo D., Zhang Y., Dou H., Chen Z. (2024). Formulating Self-Repairing Solid Electrolyte Interface *via* Dynamic Electric Double Layer for Practical Zinc Ion Batteries. Angew. Chem., Int. Ed..

[cit30] Dou H., Wu X., Xu M., Feng R., Ma Q., Luo D., Zong K., Wang X., Chen Z. (2024). Steric-hindrance Effect Tuned Ion Solvation Enabling High Performance Aqueous Zinc Ion Batteries. Angew. Chem., Int. Ed..

[cit31] Bu F., Gao Y., Zhao W., Cao Q., Deng Y., Chen J., Pu J., Yang J., Wang Y., Yang N., Meng T., Liu X., Guan C. (2024). Bio-Inspired Trace Hydroxyl-Rich Electrolyte Additives for High-Rate and Stable Zn-Ion Batteries at Low Temperatures. Angew. Chem., Int. Ed..

[cit32] Cui M., Yu L., Hu J., He S., Zhi C., Huang Y. (2025). Tailored Polymer-Inorganic Bilayer SEI with Proton Holder Feature for Aqueous Zn Metal Batteries. Angew. Chem., Int. Ed..

[cit33] Zhang S., Li Y., Bannenberg L. J., Liu M., Ganapathy S., Wagemaker M. (2024). The lasting impact of formation cycling on the Li-ion kinetics between SEI and the Li-metal anode and its correlation with efficiency. Sci. Adv..

[cit34] Wang Q., He J., Sun B., Bai Y., Yan Y., Xue J., Sun Z., Wang X., Wu J., Wang J., Zhao R., Sun Z., Liu H. K., Dou S. X. (2025). Recent Advances and Strategies of Metal Sulfides for Accelerating Polysulfide Redox and Regulating Li Plating. ACS Nano.

[cit35] Li C., Zhang X., Qu G., Zhao S., Qin H., Li D., Li N., Wang C., Xu X. (2024). Highly Reversible Zn Metal Anode Securing by Functional Electrolyte Modulation. Adv. Energy Mater..

[cit36] Li H., Ren Y., Zhu Y., Tian J., Sun X., Sheng C., He P., Guo S., Zhou H. (2023). A Bio-Inspired Trehalose Additive for Reversible Zinc Anodes with Improved Stability and Kinetics. Angew. Chem., Int. Ed..

[cit37] Yao L., Liu J., Zhang F., Wen B., Chi X., Liu Y. (2024). Reconstruction of zinc-metal battery solvation structures operating from -50 ∼ +100 degrees C. Nat. Commun..

[cit38] Yang S., Chen A., Tang Z., Wu Z., Li P., Wang Y., Wang X., Jin X., Bai S., Zhi C. (2024). Regulating the electrochemical reduction kinetics by the steric hindrance effect for a robust Zn metal anode. Energy Environ. Sci..

[cit39] Ren K., Li M., Wang Q., Liu B., Sun C., Yuan B., Lai C., Jiao L., Wang C. (2024). Thioacetamide Additive Homogenizing Zn Deposition Revealed by *In Situ* Digital Holography for Advanced Zn Ion Batteries. Nano-micro Lett..

[cit40] Wang L., Zhang B., Zhou W., Zhao Z., Liu X., Zhao R., Sun Z., Li H., Wang X., Zhang T., Jin H., Li W., Elzatahry A., Hassan Y., Fan H. J., Zhao D., Chao D. (2024). Tandem Chemistry with Janus Mesopores Accelerator for Efficient Aqueous Batteries. J. Am. Chem. Soc..

[cit41] Miki A., Nishikawa K., Ozawa T., Matsushima H., Ueda M. (2020). In Situ Measurement of Al3+ Concentration Profile during Al Anodization using Digital Holographic Interferometric Microscope. J. Electrochem. Soc..

[cit42] Zhang B., Fan H. J. (2025). Overlooked calendar issues of aqueous zinc metal batteries. Joule.

[cit43] Jiang H., Tang L., Fu Y., Wang S., Sandstrom S. K., Scida A. M., Li G., Hoang D., Hong J. J., Chiu N.-C., Stylianou K. C., Stickle W. F., Wang D., Li J., Greaney P. A., Fang C., Ji X. (2023). Chloride electrolyte enabled practical zinc metal battery with a near-unity Coulombic efficiency. Nat. Sustain..

[cit44] Wang F., Zhang J., Lu H., Zhu H., Chen Z., Wang L., Yu J., You C., Li W., Song J., Weng Z., Yang C., Yang Q. H. (2023). Production of gas-releasing electrolyte-replenishing Ah-scale zinc metal pouch cells with aqueous gel electrolyte. Nat. Commun..

[cit45] Kundu D., Adams B. D., Duffort V., Vajargah S. H., Nazar L. F. (2016). A high-capacity and long-life aqueous rechargeable zinc battery using a metal oxide intercalation cathode. Nat. Energy.

[cit46] Liu Z., Wang R., Ma Q., Wan J., Zhang S., Zhang L., Li H., Luo Q., Wu J., Zhou T., Mao J., Zhang L., Zhang C., Guo Z. (2024). A Dual-Functional Organic Electrolyte Additive with Regulating Suitable Overpotential for Building Highly Reversible Aqueous Zinc Ion Batteries. Adv. Funct. Mater..

[cit47] Lv Y., Zhao M., Du Y., Kang Y., Xiao Y., Chen S. (2022). Engineering a self-adaptive electric double layer on both electrodes for high-performance zinc metal batteries. Energy Environ. Sci..

[cit48] Ouyang K., Chen S., Ling W., Cui M., Ma Q., Zhang K., Zhang P., Huang Y. (2023). Synergistic Modulation of In-Situ Hybrid Interface Construction and pH Buffering Enabled Ultra-Stable Zinc Anode at High Current Density and Areal Capacity. Angew. Chem., Int. Ed..

[cit49] Jiao Z., Cai X., Wang X., Li Y., Bie Z., Song W. (2023). Trace Amount of Nitrilotriacetate Induced Electrolyte Evolution and Textured Surface for Stable Zn Anode. Adv. Energy Mater..

[cit50] Wan J., Wang R., Liu Z., Zhang L., Liang F., Zhou T., Zhang S., Zhang L., Lu Q., Zhang C., Guo Z. (2023). A Double-Functional Additive Containing Nucleophilic Groups for High-Performance Zn-Ion Batteries. ACS Nano.

[cit51] Li Y., Cheng J., Zhao D., Chen X., Sun G., Qiao S., Zhang W., Zhu Q. (2023). Inhibiting zinc dendrites and side reactions enabled by solvation structure regulation and facile de-solvation process. Energy Storage Mater..

