# Supplementary material for: Concentration-function coupled electrolytes harmonize thermodynamics and kinetics for stable zinc metal batteries
Source: Chem Sci. 2025 Aug 27;16(37):17426–35. doi: 10.1039/d5sc05421d (PMC12403028; doi:10.1039/d5sc05421d)
Supplement: SC-016-D5SC05421D-s001 [file SC-016-D5SC05421D-s001.pdf]

Supplementary Information (SI) for Chemical Science.
This journal is © The Royal Society of Chemistry 2025

Electronic supplementary information (ESI)

Concentration-Function Coupled Electrolytes Harmonize Thermodynamics and Kinetics for Stable Zinc Metal Batteries

Tao Liu[a], Xusheng Dong[a], Jiashuo Zhang[a], Huihui Chen[a], Rongrong Cao[a], Zixu Sun[b], Wanhai Zhou[c], Hongpeng Li[d], Dongliang Chao[c], Zhen Zhou[a*], and Ruizheng Zhao[ae*]

[a] Interdisciplinary Research Center for Sustainable Energy Science and Engineering (IRC4SE^2), Engineering Research Center of Advanced Functional Material Manufacturing of Ministry of Education, School of Chemical Engineering, Zhengzhou University, Zhengzhou 450001, Zhengzhou, China.

[b] School of Nanoscience and Materials Engineering, Henan University, Kaifeng 475004, Kaifeng, China

[c] State Key Laboratory of Molecular Engineering of Polymers and School of Chemistry and Materials, Fudan University, Shanghai 200433, Shanghai, China

[d] College of Mechanical Engineering, Yangzhou University, Yangzhou 225127, Yangzhou, China

[e] Key Laboratory of Advanced Energy Materials Chemistry (Ministry of Education), Nankai University, Tianjin 300071, Tianjin, China

[*]Corresponding author: rzzhao@zzu.edu.cn (R. Zhao); zhenzhou@zzu.edu.cn (Z. Zhou);

Experimental Section

Electrolyte Preparation

The 2 M $ZnSO_4$ (ZS) electrolyte was prepared by dissolving zinc salt ($ZnSO_4 \cdot 7H_2O$) in deionized (DI) water. The ZS-*x*Ace (*x* = 3, 6, 9, 12 M) electrolytes were prepared by adding different amounts of Ace (C_2H_5NO, AR, 99%, Aladdin) to the 2 M ZS electrolyte. ZS-*x*Asn (*x* = 0.10, 0.15, 0.20, 0.25 M) electrolytes were formed by adding different amounts of L-Asn ($C_4H_8N_2O_3$, 98%, Aladdin) to 2 M ZS electrolytes. ZS-9Ace-*x*Asn (*x* = 0.10, 0.15, 0.20, 0.25 M) electrolytes were formed by adding different amounts of Asn to ZS-9Ace. The final optimized amounts of 9 M Ace and 0.2 M Asn are referred to as ZS-Dual. ZS-9Ace and ZS-0.2Asn will be referred to as ZS-Ace and ZS-Asn in subsequent control experiments.

Electrode Preparation

The purchased zinc foils (thickness 100 µm and 50 µm, 99.99 %) were polished with sandpaper to remove surface impurities. The zinc foils were cut into slices of 12 mm diameter discs, washed several times with dilute hydrochloric acid (HCl, 12 vol %) and ethanol (C_2H_5OH, AR, 99.9% Innochem) to remove the surface oxide passivation layer, and dried under vacuum at 60 °C for 12 h.

The synthesis of $Zn_xV_2O_5 \cdot nH_2O$ (ZVO) was carried out by hydrothermal method. Specifically, 0.545 g of commercial V_2O_5 and 0.44 g of $Zn(AC)_2$ were dissolved in 70 ml DI water, then 5 ml acetone and 2 ml 10 wt% HNO_3 were added to the solution under wet ultrasonication for 10 min. Finally, the mixture was transferred to a 100 ml Teflon-lined stainless-steel autoclave and kept in an oven at 180 °C for 24 h. After cooling, the product was collected and washed repeatedly with DI water, and then dried under vacuum at 80 °C for 12 h. The obtained ZVO powder was mixed with acetylene black and polyvinylidene fluoride (PTFE) binder in isopropyl alcohol according to a mass ratio of 7:2:1, and uniformly ground to a slurry. The slurry was then rolled into raw films, which were then punched into 12 mm diameter discs, pressed onto the Ti mesh and dried in a vacuum oven at 80 °C overnight. The mass loading of the cathode was approximately 2.5 mg cm^{-2}.

Characterization

Differential scanning calorimetry (DSC) was performed on a NETZSCH DSC 200F3 provided by eceshi (www.eceshi.com) and samples scanned from room temperature to –100 °C at a rate of 10 °C min^{-1} under a nitrogen atmosphere. Raman and FTIR spectra were collected by In Via Qontor (Renishaw, UK) and INVENI0 FTIR

spectrophotometer (BRUKER, US). ^{1}H (H_2O) nuclear magnetic resonance (NMR) spectroscopy in electrolytes was studied with JNM-ECZ600R/S3. The phase structure of the Zn anode before and after immersion and cycling was characterized by X-ray diffraction (XRD, Rigaku Ultima IV diffractometer with Cu-Kα radiation, operating at 40 kV and 40 mA). The morphology of Zn deposits on the electrode surface was carried out by operating scanning electron microscope (JSM-7900F). Assembly of a Zn||Zn symmetric cell for differential electrochemical mass spectrometry (DEMS) testing (HPR-40, HIDEN). The electronic structure and the corresponding composition were analyzed by X-ray photoelectron spectroscopy (XPS, Thermo Scientific K-Alpha+). The component distribution of the Zn anode surface after cycling was further characterized by time-of-flight secondary ion mass spectrometry (PHI nano TOF3), which was equipped with a 2 keV Ar^{+} sputter gun. Atomic force microscopy (Bruker Dimension Icon) was used to study the morphology of the cycled Zn anode.

Electrochemical Tests

Coin battery components (CR2032, spacer: 15.8 × 1 mm, spring: 15.4 × 1.1 mm) purchased from Canrd Technology Co. Ltd. Coin batteries are prepared using a hydraulic crimping machine (MSK-110) purchased from Shenzhen Kejing Star Technology company. Galvanostatic cycling of all cells was tested using Neware battery test system (CT-4008T and CT-4008Q) and LAND test systems (CT3002A). The Zn dendrite growth process was visualized using an optical microscope (LV150N, Nikon). Chronoamperometry (CA), electrochemical impedance spectroscopy (EIS), linear scanning voltammetry (LSV) and Cyclic voltammetry (CV) were carried out on a Solartron Energylab electrochemical workstation (Ametek). The CA measurement was examined on Zn||Zn symmetric cells by applying a constant overpotential of –150 mV for 300 s. The EIS were tested at an open-circuit voltage with an amplitude of 5 mV and a frequency range of 100 kHz to 0.01 Hz. The distribution of relaxation times (DRT) analysis was performed using DRT Tools.[1] The Cyclic voltammetry (CV) curves of the Zn||Cu asymmetric cells were recorded between –0.2 V and 0.4 V at a scanning rate of 0.5 mV s^{-1}.Corrosion potential and current were assessed using Tafel plots derived from linear polarization (LP) curves at 1.0 mV s^{-1} in different electrolytes. HER curves were obtained through linear scanning voltammetry (LSV) tests.

Assembly of a Zn||Zn symmetric cell for differential electrochemical mass spectrometry (DEMS) testing (HPR-40, HIDEN). Argon gas was passed through the cell chamber at a flow rate of 0.4 mL min^{-1}, and the cells were charged and discharged after 2 h at 1 mA cm^{-2} and 0.5 mAh cm^{-2}

The desolvation activation energy (E_a) was calculated from the EIS of Zn||Zn symmetric cells at different temperatures using the Arrhenius equation:

$$\frac{1}{R_{ct}} = Aexp\left(\frac{E_a}{RT}\right)\#(2)$$

where R_{ct} is the Zn^{2+} charge transfer resistance, *A* is the frequency factor, *R* is the molar gas constant, and *T* the is Kelvin temperature. The E_a was obtained from the slope of the $ln\,R_{ct}^{-1}$ versus 1000/T Arrhenius curves.

The transference number of Zn^{2+} was measured with the EIS before and after the CA test based on the following equation:

$$t_{Zn^{2+}} = \frac{I_s(\Delta V - I_0 R_0)}{I_0(\Delta V - I_S R_S)}\#(3)$$

where ΔV is the voltage polarization (10 mV), I_0 and R_0 are the initial current and resistance, I_S and R_S are the steady state current and resistance, respectively.

The ionic conductivity is calculated from the EIS data of the SS||SS coin cells via the following formula (SS stands for stainless steel plate):

$$\sigma = \frac{L}{RS}\#(4)$$

where L is the distance between the two electrodes, S is the contact area between the electrode and the electrolyte and R is the bulk resistance in Nyquist plots.

The electric double layer capacitance (EDLC) was calculated from the equation:

$$C = \frac{i_c}{v}\#(5)$$

where $i_c = (i_{V+} - i_{V-})/2$, which is the half of the current difference between the forward scan and negative scan at 0 V. v denotes the scan rates of the CV tests. The corresponding CV was conducted by scanning between –15 and 15 mV at scan rates of 6, 8, 10, 12, 14 and 16 mV with Zn||Zn Swagelok® cells.

The differential capacitance of the EDL is calculated from the AC impedance test data of the Zn||Cu cells using the following equation:

$$C_d = -\frac{1}{2\pi f Z_{im}}\#(6)$$

where $C_d\ (\mu F\ cm^{-2})$ is differential capacitance, $f\ (Hz)$ is the frequency of the impedance test (1000Hz) and Z_{im} is the imaginary part of the impedance.

Electrochemical Digital Holography

Electrochemical digital holography was used to observe changes in the concentration of specific species at the electrode interface during electrochemical processes. The system is based on a Mach-Zehnder interferometer,

using a charge-coupled device camera to record interferograms of the electrode/electrolyte interface, which are then converted into phase maps by numerical reconstruction. Zn||Zn symmetric cells with different electrolytes were assembled, and the above principle was utilized to monitor the amplitude and phase changes at the electrode/electrolyte interface during Zn electrodeposition, revealing the dynamic evolution of the concentration gradients and the diffusion layer, which can be described as follows:

$$\Delta C = k\Delta n = k\left(\frac{\lambda_0}{2\pi d}\right)\Delta\phi \#(1)$$

where k is a proportionality constant characterizing the linear relationship between solution concentration and refractive index. λ_0 is the wavelength of the laser and d is the thickness of the solution layer of the concentration change. ΔC, Δn and $\Delta\phi$ represent the concentration change, refractive index change and phase variation at the interface, respectively. In the phase diagram:

The green region, $\Delta\phi = 0$, $\Delta C = 0$, indicates that the concentration remains constant.

The blue region, $\Delta\phi < 0$, $\Delta C < 0$, indicates a decrease in concentration

The yellow or red region, $\Delta\phi > 0$, $\Delta C > 0$, indicates an increase in concentration.

Computational methods

First-principles calculations were carried out based on the density functional theory (DFT). The structure optimization and free energy calculations of were conducted by applying the Vienna Ab initio Simulation Package (VASP) code.[2-4] The Projector Augmented Wave (PAW) method could be conducted considering spin-polarized calculations.[5] The exchange correlation functional could be investigated by Perdew−Burke−Ernzerhof (PBE) type of generalized gradient approximation (GGA). A plane wave basis set with an energy cutoff of 450 eV could be served throughout the calculations.[6] The K-point mesh of 3 × 3 × 1 was applied to the supercell of Zn surface model, and 3 × 3 × 3 was used for molecule interaction model. The structure was optimized until the force convergence was 0.01 eV/Å, and the energy convergence was 10^{-5} eV in this work. To analyze the interaction quantitatively, the energy is estimated by the following equation:

$$E = E_{a+b} - E_a * E_b \#(7)$$

where E stands for the free energy, the E_{a+b} is the total energy of the model, and E_a, E_b represent the energy of the monomer models in different relaxation systems.

Molecular dynamics simulations were carried out using the Large-scale Atomic/Molecular Massively Parallel Simulator (LAMMPS).[7] The force-fields parameters of Zn^{2+} and sulfate were taken from previous publications and

a charge scaling of 0.8 was adopted to mimic polarization and charge transfer effects.[8, 9] The force-fields parameters of Ace and Asn were obtained from the LigParGen web server.[10] The tip3p water model was adopted. The systems were initially set up using PACKMOL and Moltemplate software.[11, 12] Periodic boundary conditions (PBCs) were applied in all three directions. A cutoff of 12 Å was used for both van der Waals interactions and the long-range correction of the particle–particle particle-mesh of the Coulombic interactions.

For the solvation structure, an initial energy minimization was performed at 0 K (energy and force tolerances of 10^{-6}) to obtain the ground-state structure. Then the simulations were started with a 200 ps NVT from 0 K to 298 K. Then, the systems were equilibrated at 298 K and 1 atm in the NPT ensemble for 20 ns. Finally, the production run was performed in the NPT ensemble for 5 ns. The solvation structure is obtained and analyzed during the production run. VMD software is used to visualize the snapshots and analyze the results. The percentage of different solvation structures is analyzed by some Python scripts written by ourselves. A time step of 1 fs was used for all simulations.

For the electrochemical double layer simulations, two four-layer zinc slabs were added to represent the positive and negative electrodes. To reduce the Coulomb effect between the mirrored slabs due to the periodic boundary condition, a 6 nm thick layer of vacuum was extended along the z axis. The potential difference was controlled by the constant potential method (CPM) using the ELECTRODE package in LAMMPS.[13] An initial energy minimization at 0 K (energy and force tolerances of 10^{-6}) was performed to obtain the ground-state structure. The simulations were then started with a 200 ps NVT from 0 K to 298 K. The systems were equilibrated at 298 K in the NVT ensemble for 3 ns. Finally, the production run was performed in the NVT ensemble for 2 ns. The density distribution is obtained and analyzed during the production run. VMD software is used to visualize the snapshots and analyze the results. A time step of 1 fs was used for all simulations.

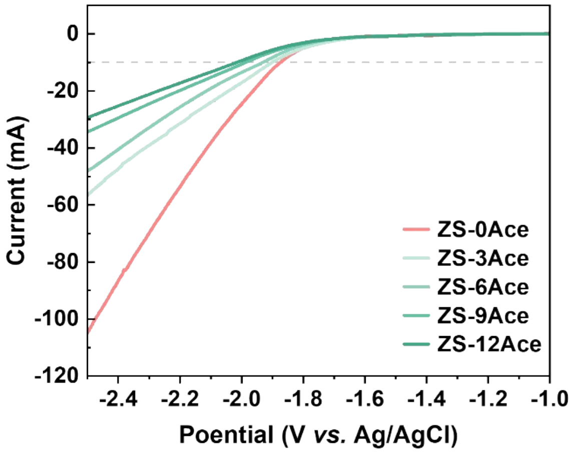

Fig. S1. LSV curves in different electrolytes for Zn anodes.

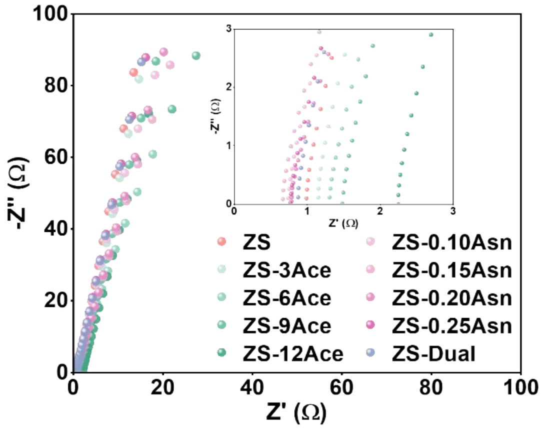

Fig. S2. EIS curves of SS||SS coin cells for different electrolytes.

According to the ionic conductivity equation, a lower bulk resistance *R* implies a higher conductivity. From the EIS plot, it can be deduced that Asn has the ability to enhance the conductivity and is therefore expected to mitigate the sacrificed kinetics due to Ace.

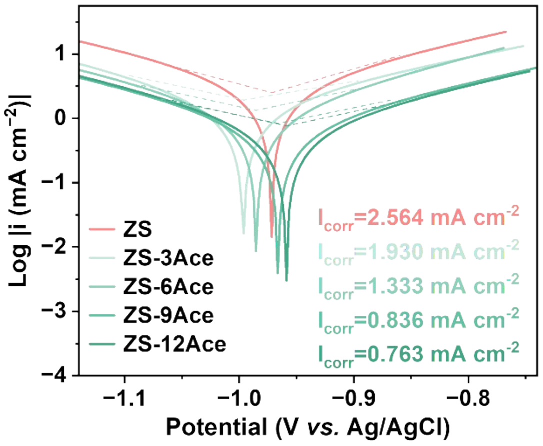

Fig. S3. Tafel plots of the Zn plate tested in ZS-*x*Ace electrolytes.

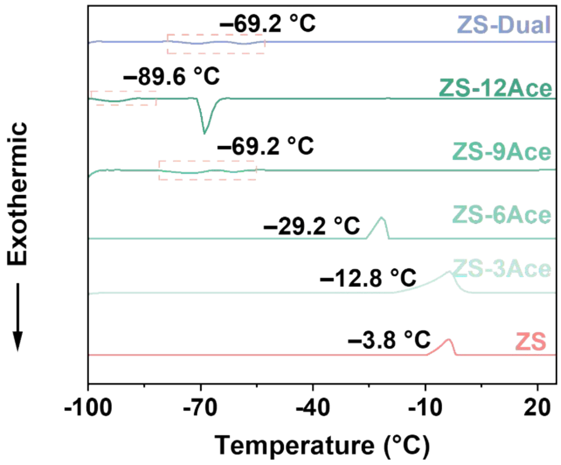

Fig. S4. Differential scanning calorimetry (DSC) curves for ZS-xAce and ZS-Dual electrolytes.

DSC was used to study the phase transition behavior. ZS, ZS-3Ace, and ZS-6Ace exhibited distinct endothermic peaks corresponding to solid to liquid transitions. In contrast, higher concentrations of Ace did not show a clear melting peak but instead a gradual signal indicative of a glass transition.

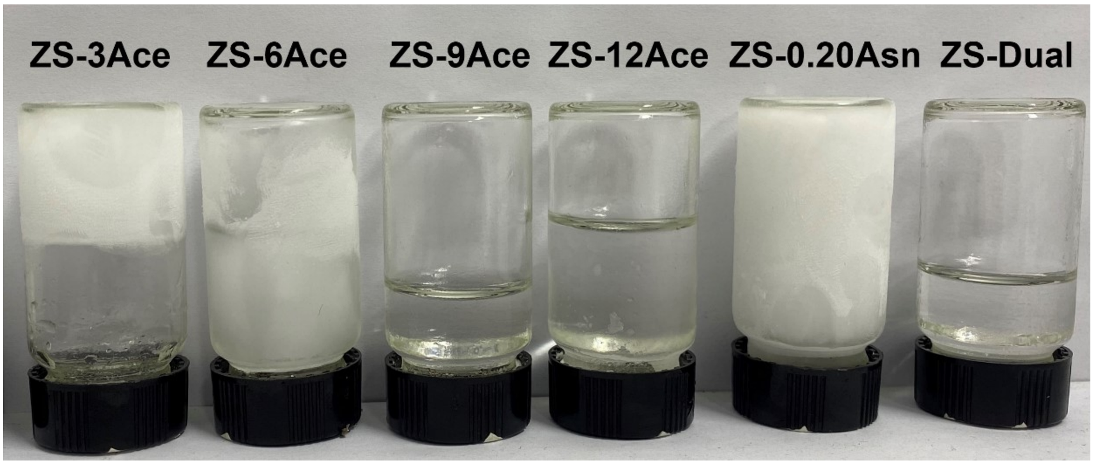

Fig. S5. Anti-freezing experiment with different electrolytes at −25°C.

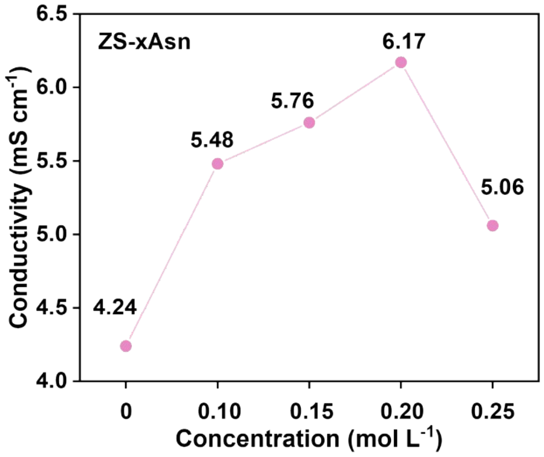

Fig. S6. Ionic conductivity of ZS-*x*Asn (*x* = 0, 0.10, 0.15, 0.20, 0.25 M) electrolytes.

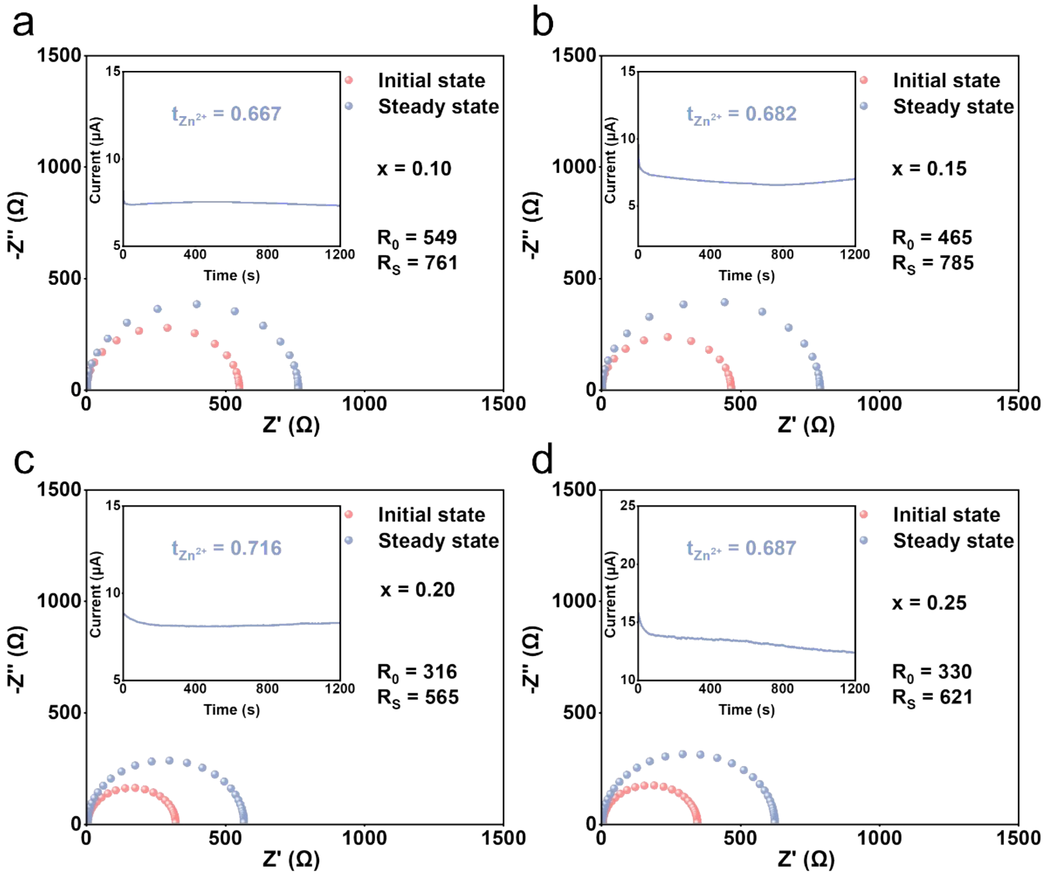

Fig. S7. Nyquist plots before and after polarization and CA curves for symmetric cells in ZS-9Ace-*x*Asn electrolytes, *x* = (a) 0.10, (b) 0.15, (c) 0.20 and (d) 0.25.

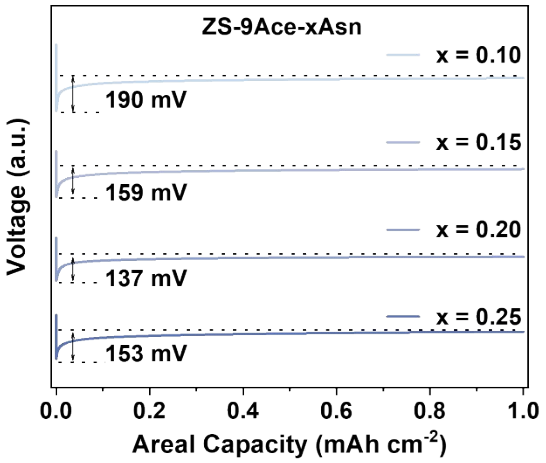

Fig. S8. Polarization curves of Zn deposition of a Zn||Zn symmetric cell at 10 mA cm^{-2} , 1 mAh cm^{-2} in different electrolytes.

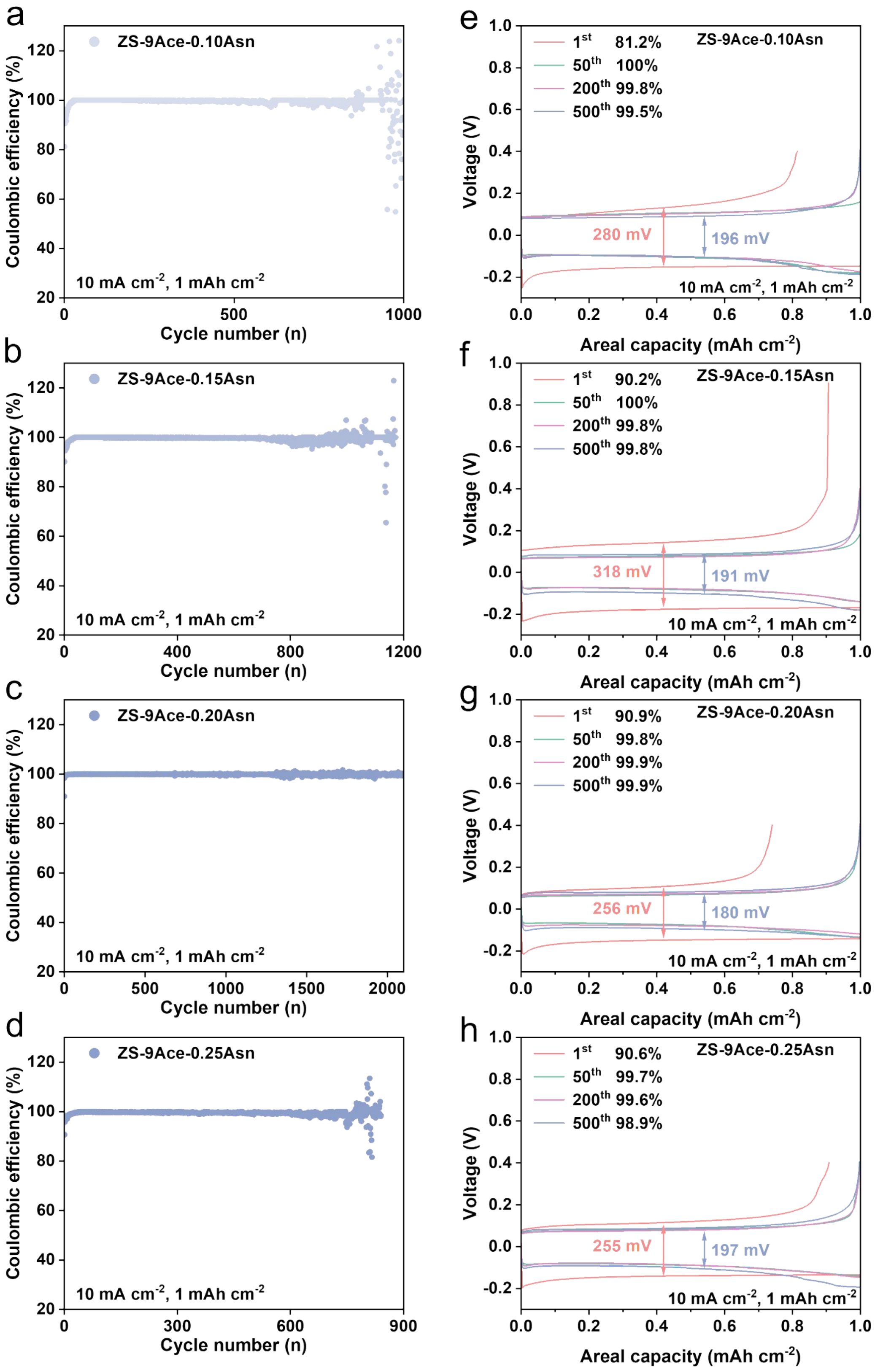

Fig. S9. (a-d) Zn plating/stripping CE in a Zn||Cu asymmetric cell at 2 mA cm^{-2} ,1 mAh cm^{-2}, and (e-h) the corresponding galvanostatic voltage profiles at different cycles in different electrolytes.

To screen the optimal ratio of the hybrid electrolyte, the CE and polarization of the Zn||Cu asymmetric cell using

ZS-9Ace-*x*Asn electrolyte were investigated. As shown, the cell using ZS-9Ace-0.2Asn electrolyte demonstrated exceptional performance, achieving a cycle of 2000 times at 10 mA cm^{-2} with 99.9% initial CE and 99.8% average CE, which is far superior to other formulations. Furthermore, the polarization voltage of 255 mV for the first cycle and 180 mV for the 500th cycle were the lowest. ZS-9Ace-0.2Asn (ZS-Dual) was selected as the optimal hybrid electrolyte based on comprehensive analysis of ionic conductivity, Zn||Zn symmetric cell, and Zn||Cu asymmetric cell.

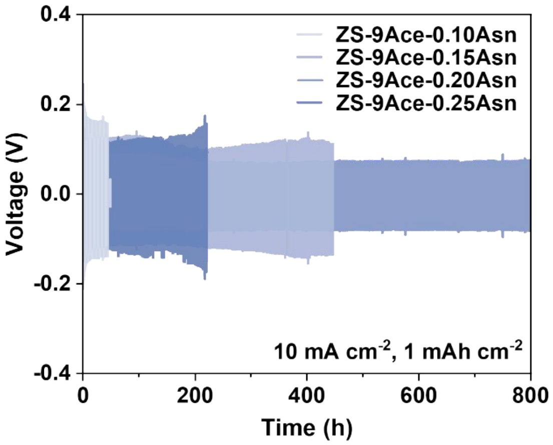

Fig. S10. Galvanostatic cycling performance of a Zn||Zn symmetric cell at 10 mA cm^{-2} , 1 mAh cm^{-2} in different electrolytes.

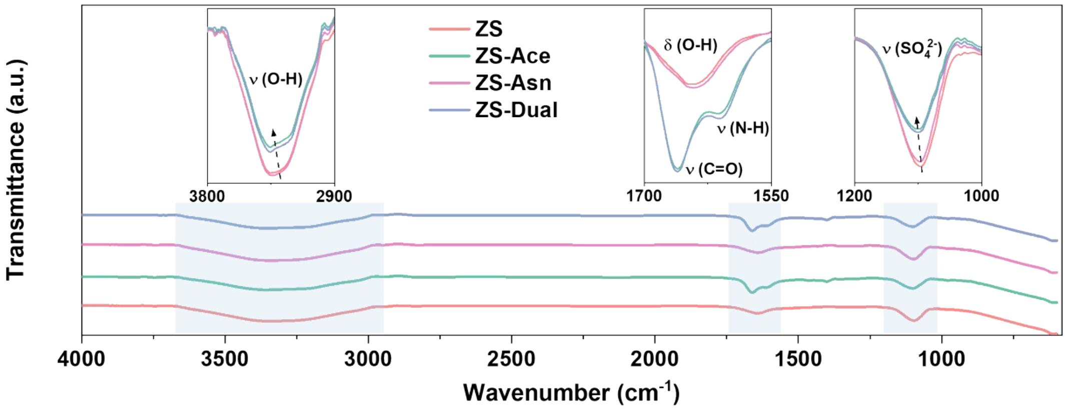

Fig. S11. FTIR spectra of different electrolyte.

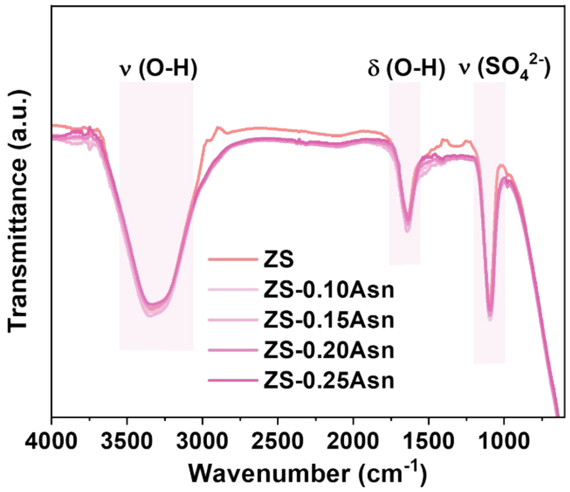

Fig. S12. FTIR spectra of ZS-*x*Asn (*x*= 0.10, 0.15, 0.20, 0.25)

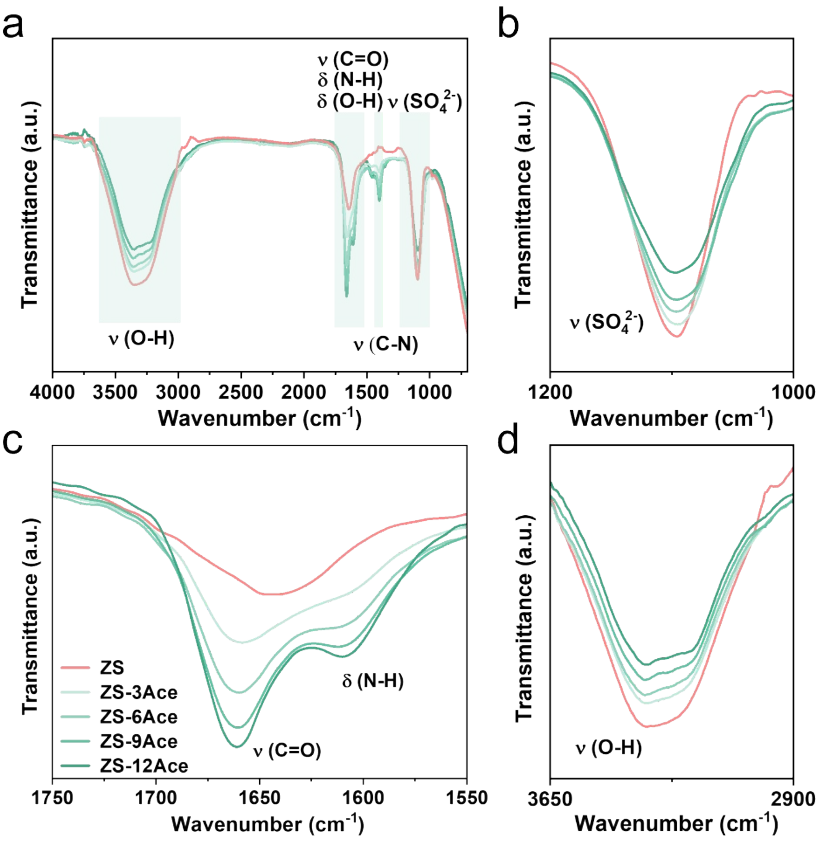

Fig. S13. (a-d) FTIR spectra of ZS-*x*Ace (*x*= 0, 3, 6, 9, 12 M).

With the increase of Ace concentration, the hydrogen bond interaction between N-H and water as well as the coordination effect between C=O and Zn^{2+} were gradually enhanced. As a result, the Ace-containing electrolyte exhibited separated peaks for C=O and N-H compared to the single peak observed at 1640 cm^{-1} in ZS.

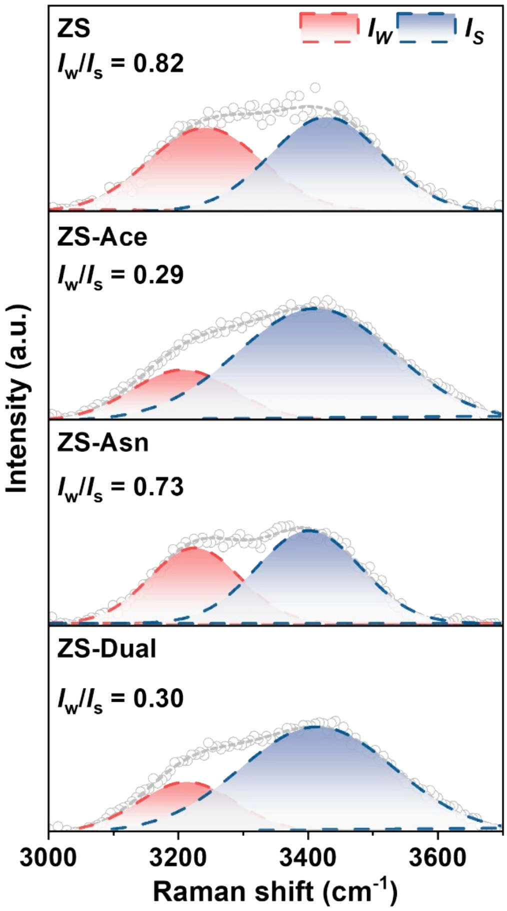

Fig. S14. Fitted Raman spectra of different electrolytes.

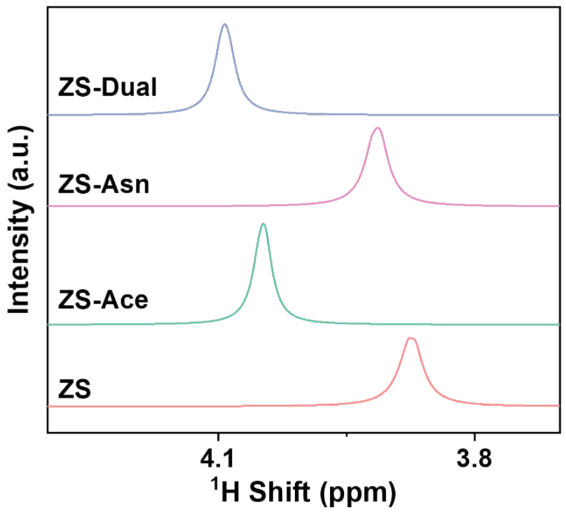

Fig. S15. The ^1H NMR spectra of different electrolytes.

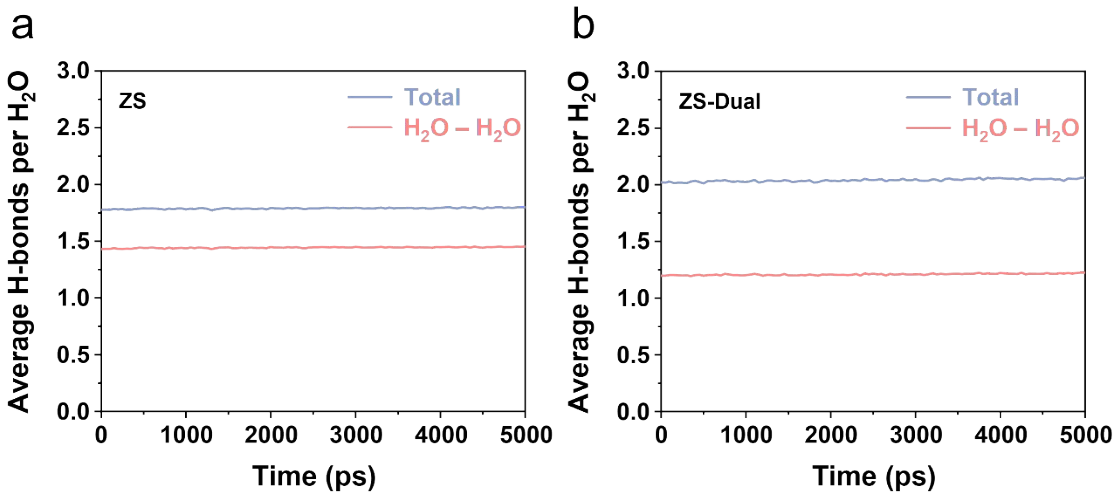

Fig. S16. Average number of HBs of water molecules in the **a** ZS and **b** ZS-Dual electrolyte.

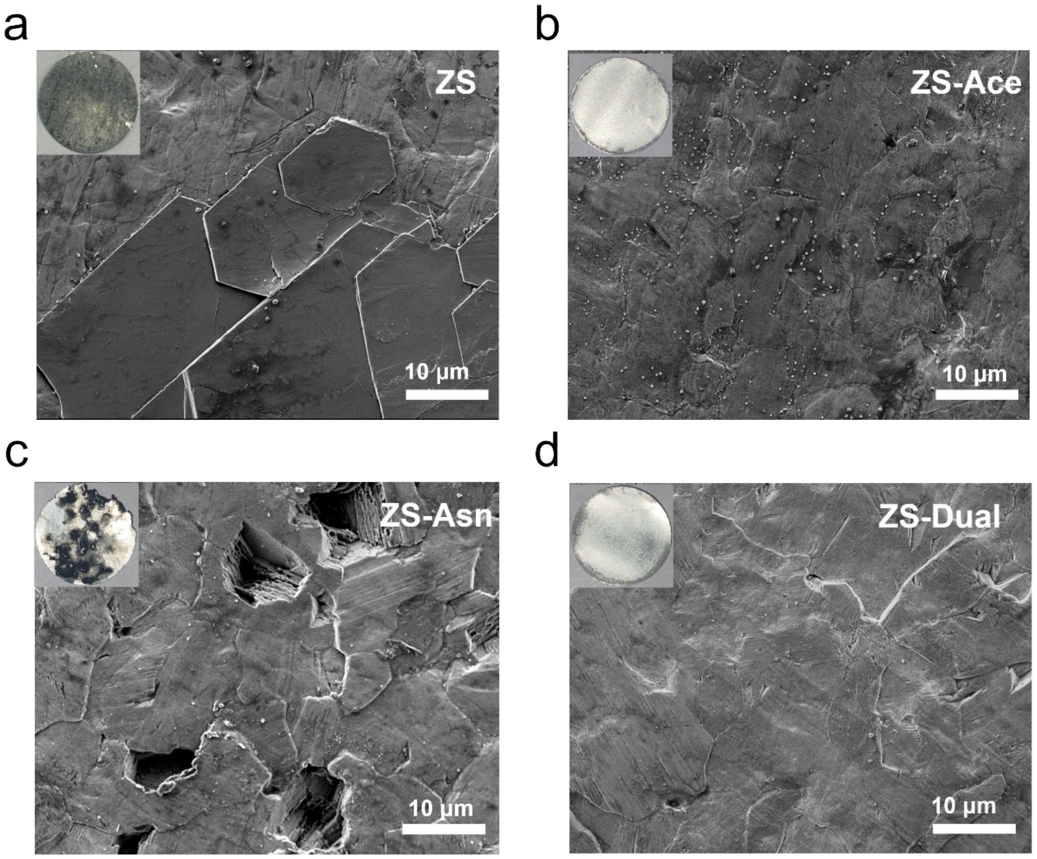

Fig. S17. Planar SEM of Zn anodes after 7-day immersion in the (a) ZS, (b) ZS-Ace, (c) ZS-Asn, and (d) ZS-Dual electrolyte..

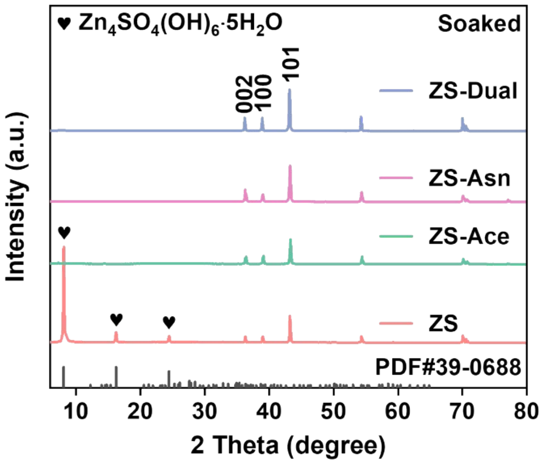

Fig. S18. XRD patterns of Zn anodes after 7-day immersion in different electrolytes.

The XRD patterns of the Zn foils after immersion in ZS exhibit a noticeable by-product signal, which corresponds to the hexagonal plates scattered on the surface of the Zn anode in **Fig. S17a**. No such alkaline by-product signal is seen in the other three groups.

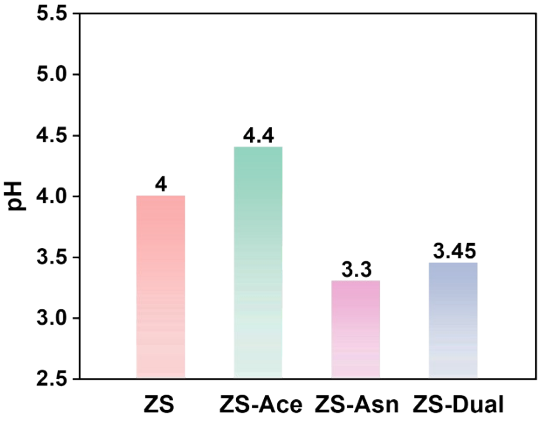

Fig. S19. pH of different electrolytes.

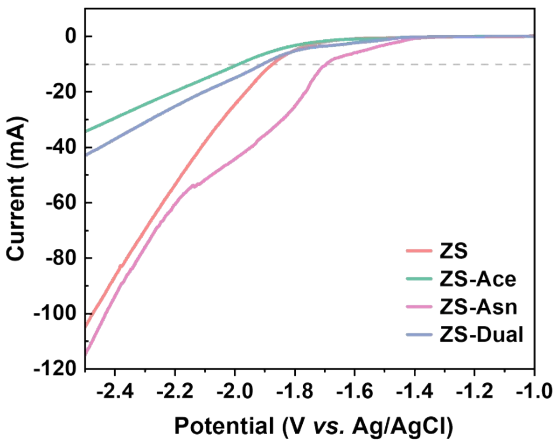

Fig. S20. LSV curves in different electrolytes for Zn anodes.

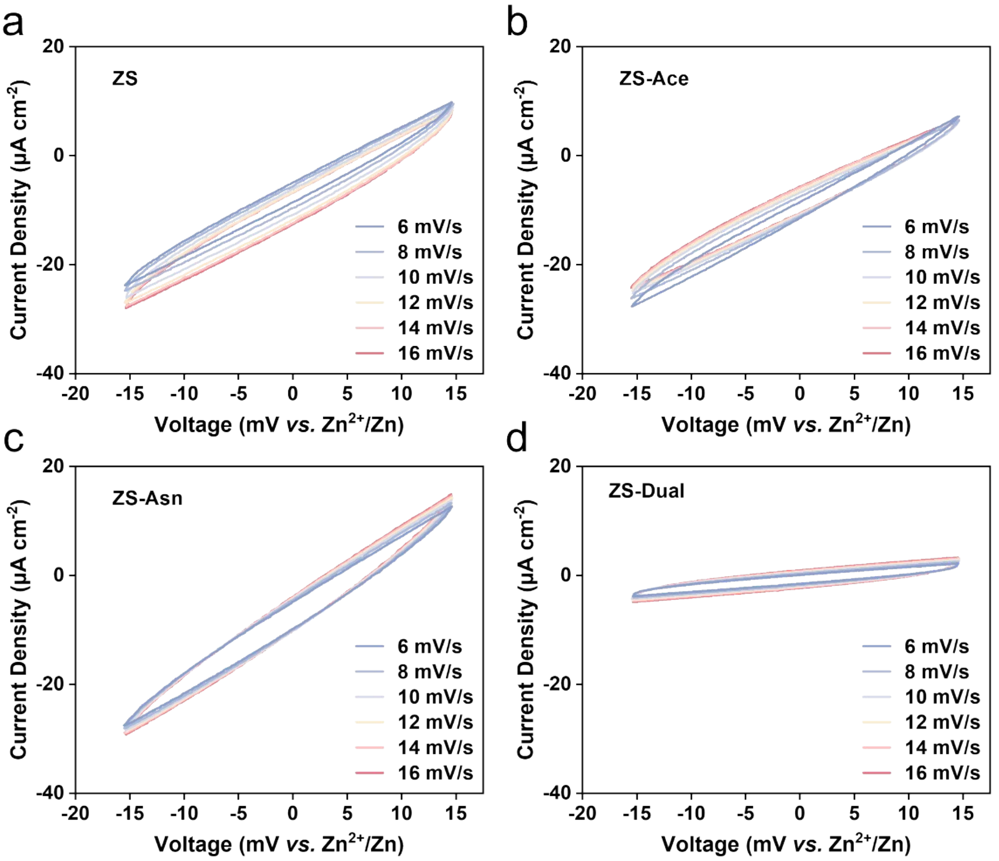

Fig. S21. CV curves of Zn||Zn symmetric cells in the voltage range from -15 mV to 15 mV at various scan rates in (a) ZS, (b) ZS-Ace, (c) ZS-Asn, and (d) ZS-Dual electrolyte.

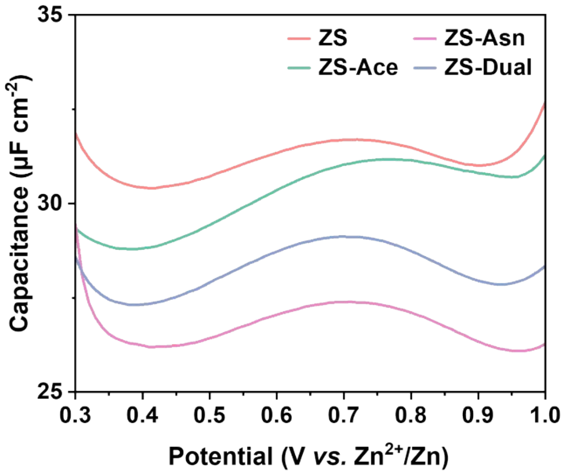

Fig. S22. Differential capacitance curves of Zn||Cu asymmetric cells in different electrolytes.

The capacitance values of different samples follow the same order with ELDC, which further proves the adsorption of Asn in IHP.

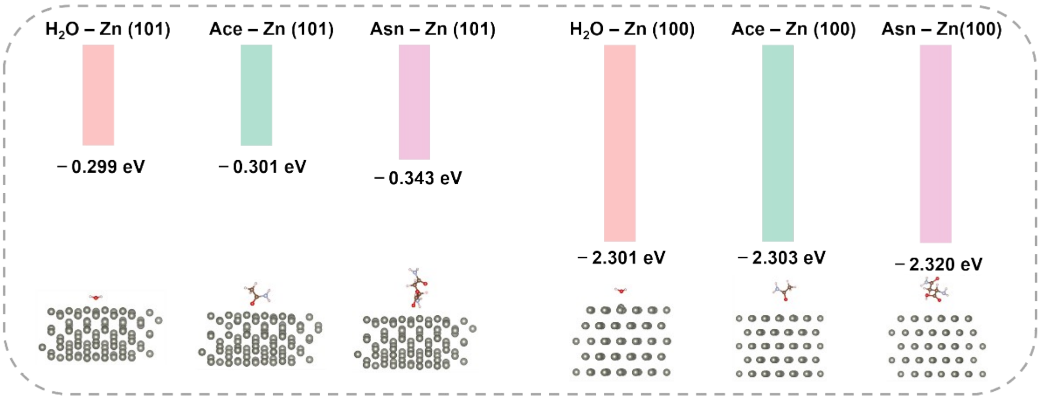

Fig. S23. Comparison of the adsorption energy of different molecules on Zn(101) and (100) slab.

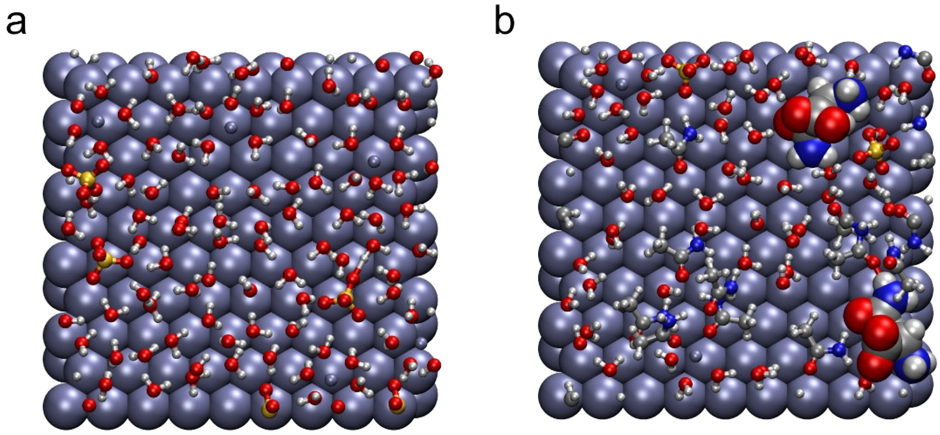

Fig. S24. Snapshots of MD simulations on electric double layers for (a) ZS, and (b) ZS-Dual at the Zn anode (top view).

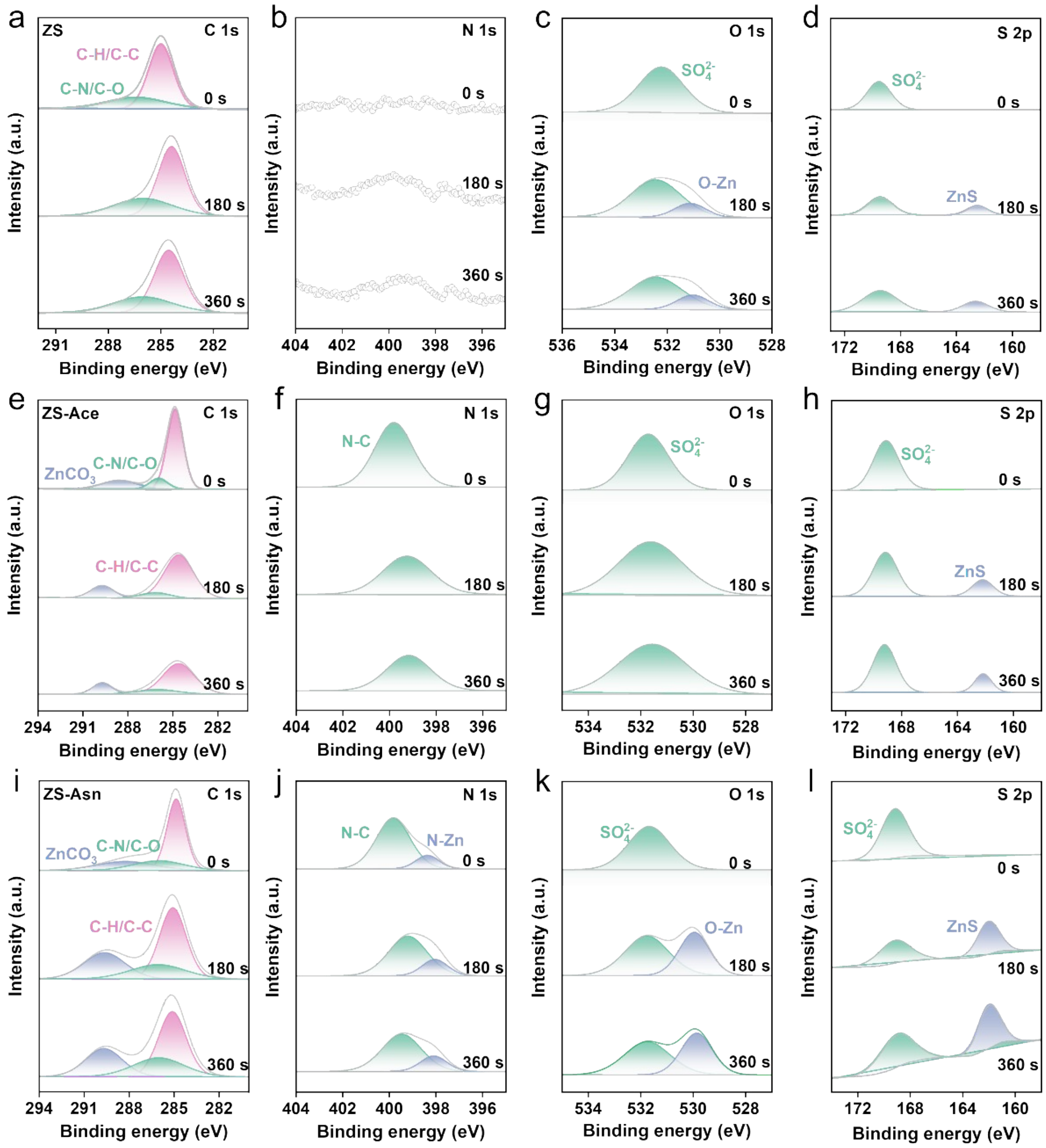

Fig. S25. XPS with in-depth profiles of the Zn anode after 50 cycles with different Ar^+ sputtering times (0, 180 and 360 s) in (a-d) ZS, (e-h) ZS-Ace, and (i-l) ZS-Asn.

XPS analysis of the Zn anode after 50 cycles in different electrolytes showed that $ZnCO_3$, N-C and ZnS in ZS-Dual were induced by both Ace and Asn. N-Zn and O-Zn were specifically attributed to Asn, while $ZnSO_3$ was induced by Ace.

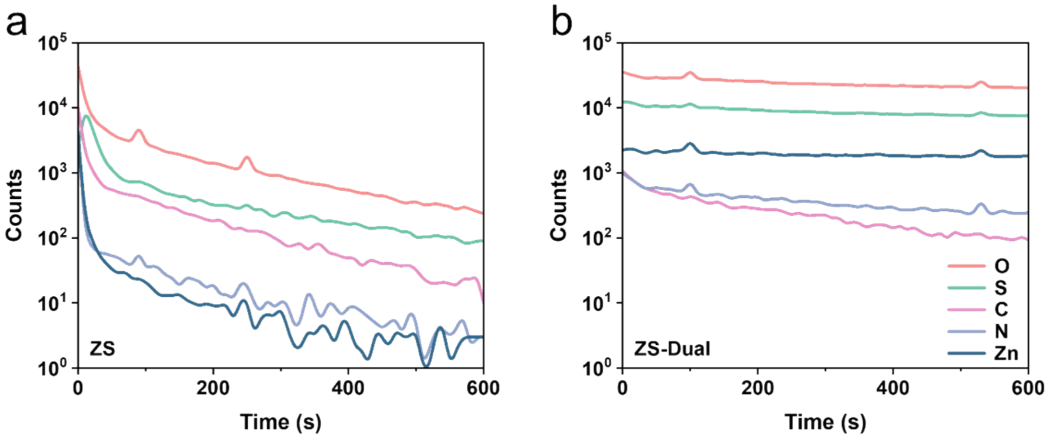

Fig. S26. Depth profile curves of O, S, C, N and Zn secondary ions over 600 s etching time for the Zn anode after 50 cycles in (a) ZS, and (b) ZS-Dual electrolytes.

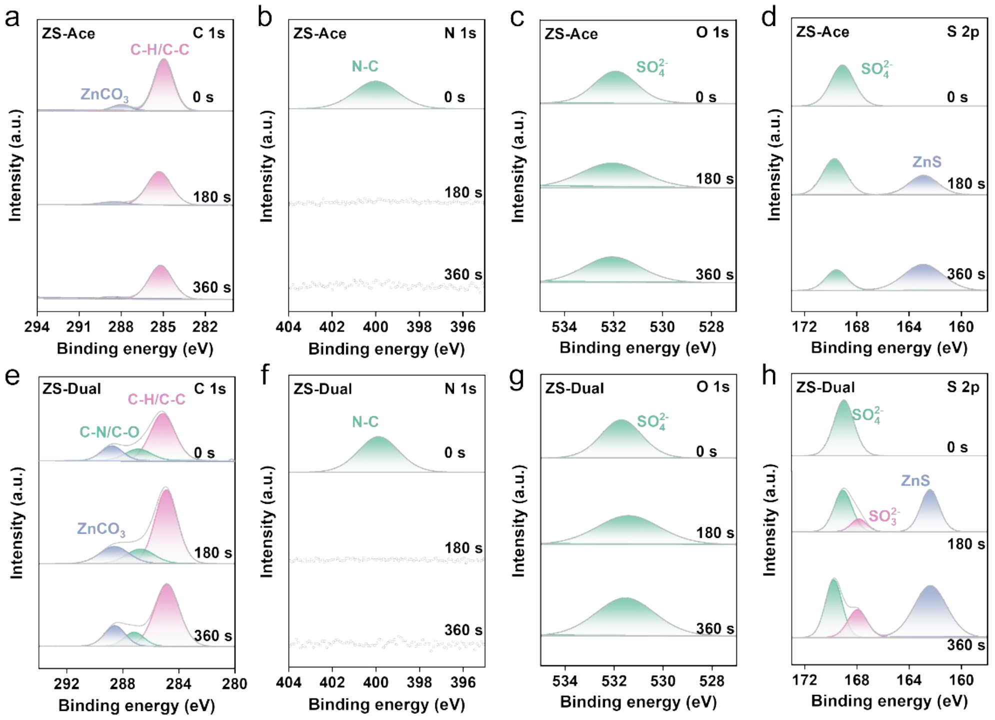

Fig. S27. XPS with in-depth profiles of the Zn anode after 50 cycles with different Ar^+ sputtering times (0, 180 and 360 s) in (a-d) ZS-Ace, and (e-h) ZS-Dual under –25°C.

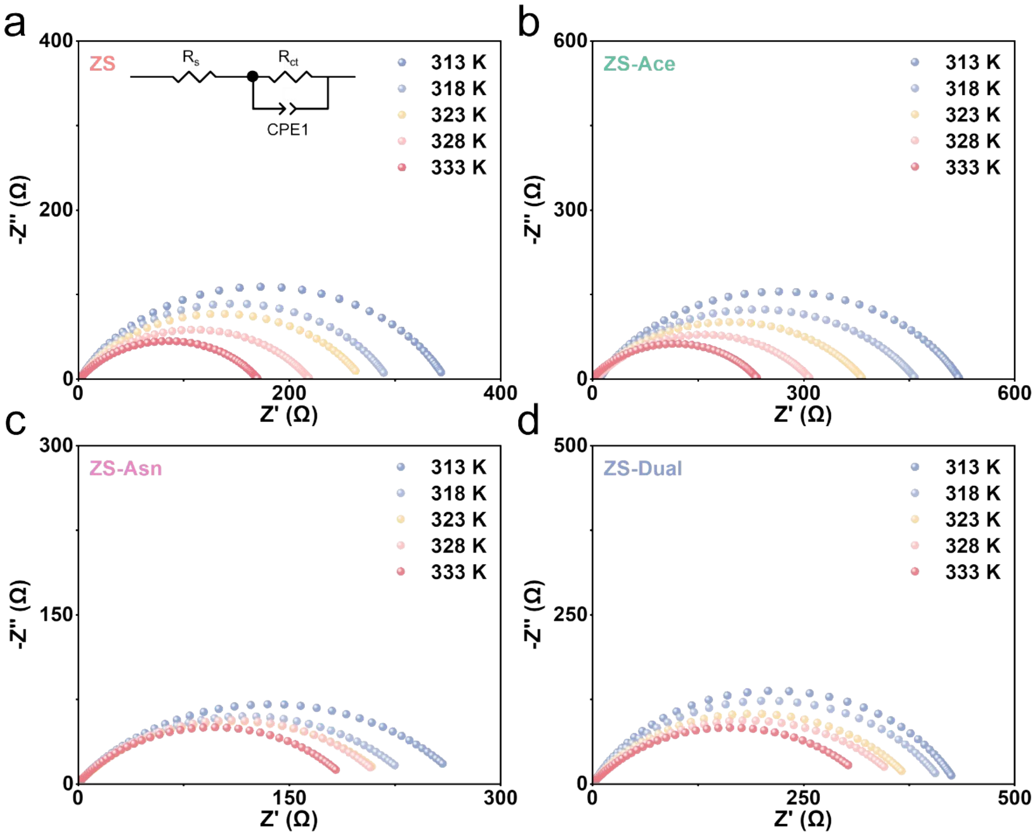

Fig. S28. Nyquist plots before corresponding to different temeratures for symmetric cells using (a) ZS, (b) ZS-Ace, (c) ZS-Asn, and (d) ZS-Dual electrolyte..

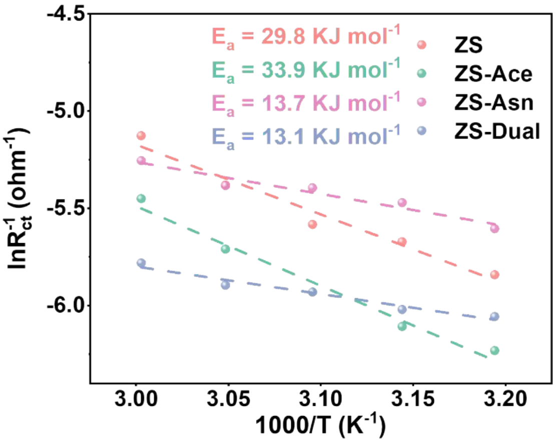

Fig. S29. Activation energy E_a based on the fitting of ln R_{ct}^{-1} versus 1000/T Arrhenius curves in different electrolytes.

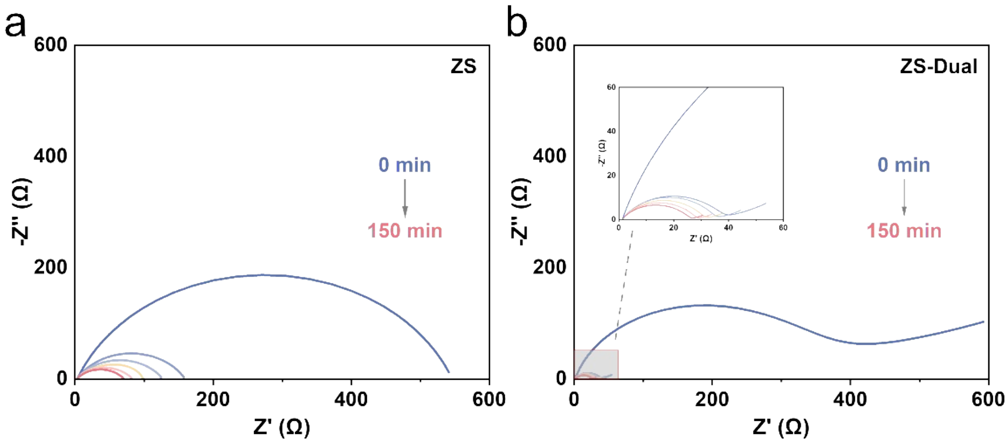

Fig. S30. In situ EIS tests for Zn anode in (a) ZS and (b) ZS-Dual electrolytes.

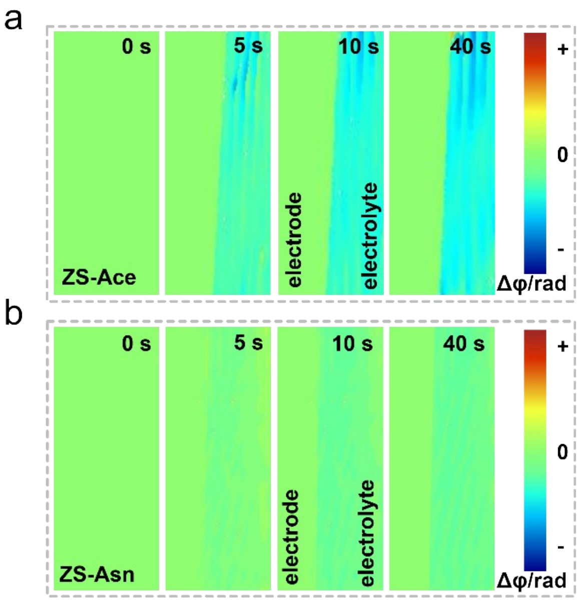

Fig. S31. *In situ* EDH of the evolution of the Zn^{2+} concentration at the electrode interface in (a) ZS-Ace, and (b) ZS-Asn electrolytes.

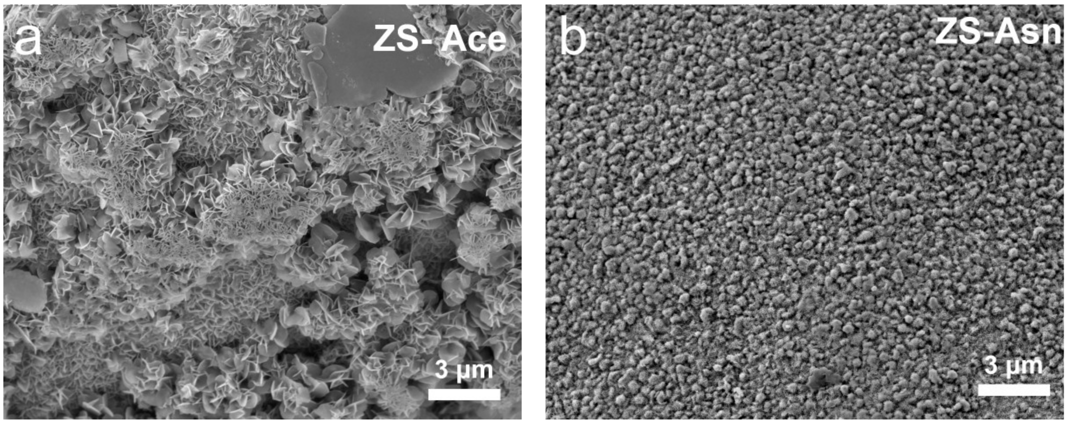

Fig. S32. Planar SEM of Zn deposits with 1 mAh cm^{-2} in (a) ZS-Ace, and (b) ZS-Asn electrolytes.

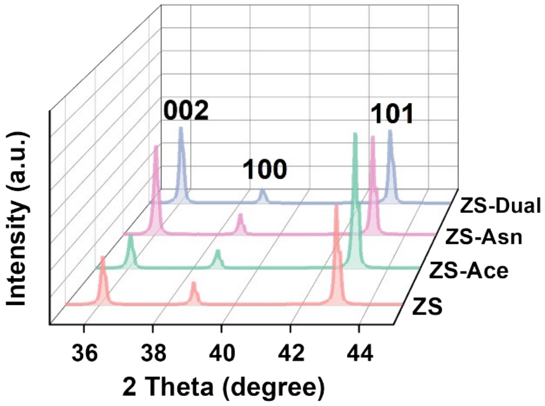

Fig. S33. XRD patterns of Zn anodes cycled at 5 mA cm^{-2}, 1 mAh cm^{-2} with different electrolytes.

To visualize the Zn texture evolution, the relative texture coefficients (RTC) of three typical Zn crystal planes were calculated by the following equation:

$$RCT_{(hkl)} = \frac{I_{(hkl)}/I_{0(hkl)}}{\Sigma\left(I_{(hkl)}/I_{0(hkl)}\right)} \times 100\#(1)$$

where $I_{(hkl)}$ and $I_{0(hkl)}$ are the peak intensities obtained from the XRD patterns of the samples and standard Zn (PDF#870713), respectively. Higher RTC values indicate a greater tendency to crystal texturing.[14, 15]

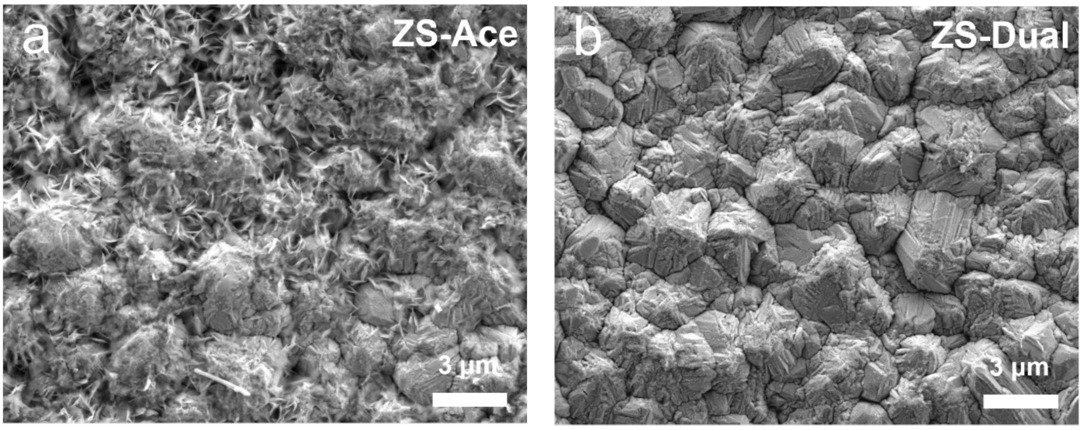

Fig. S34. Planar SEM of Zn deposits with 1 mAh cm^{-2} in (a) ZS-Ace, and (b) ZS-Asn electrolytes under –25°C.

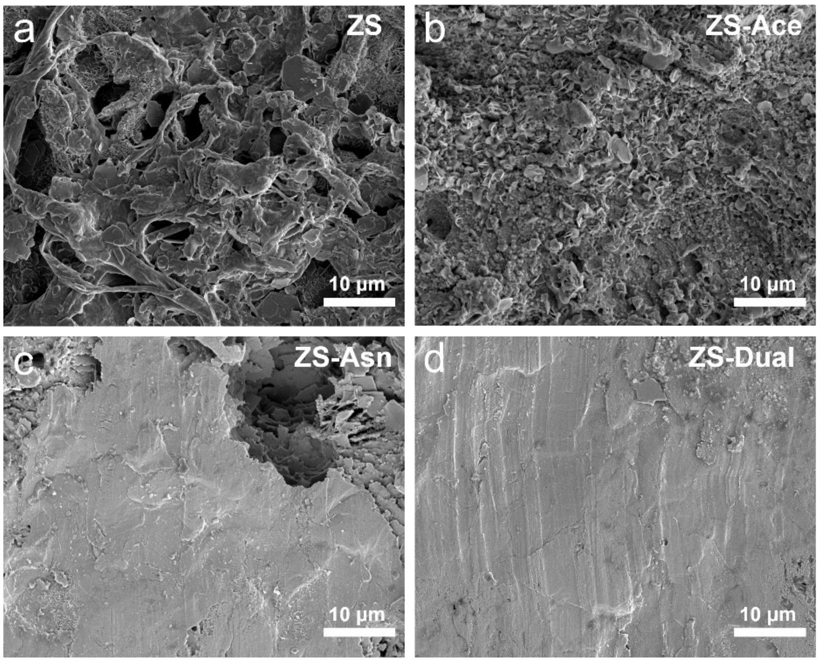

Fig. S35. Planar SEM of the Zn anode after 50 cycles in (a) ZS, (b) ZS-Ace, (c) ZS-Asn, and (d) ZS-Dual electrolyte.

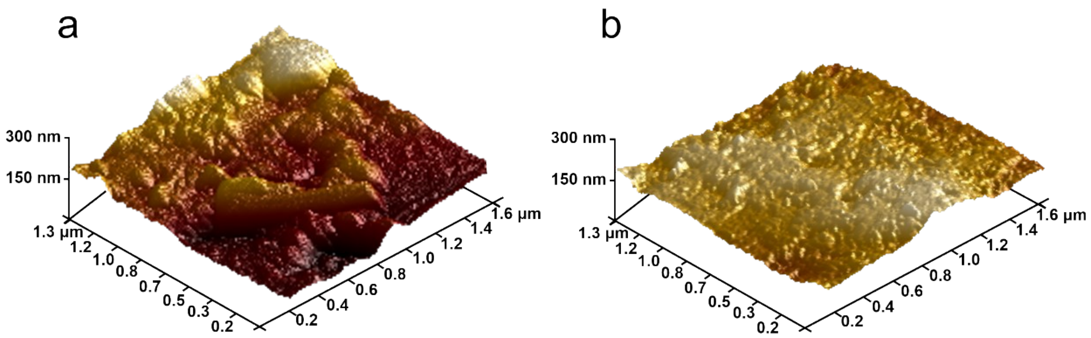

Fig. S36. AFM images of the Zn anode after 50 cycles in (a) ZS, and (b) ZS-Dual electrolytes.

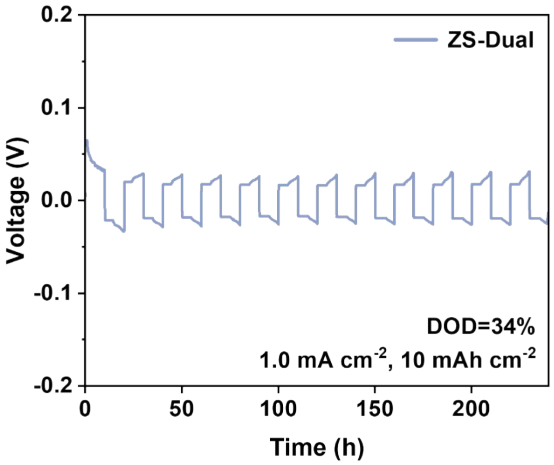

Fig. S37. Cycling stability of Zn||Zn symmetric cells under 34% depth of discharge (thickness 50 μm).

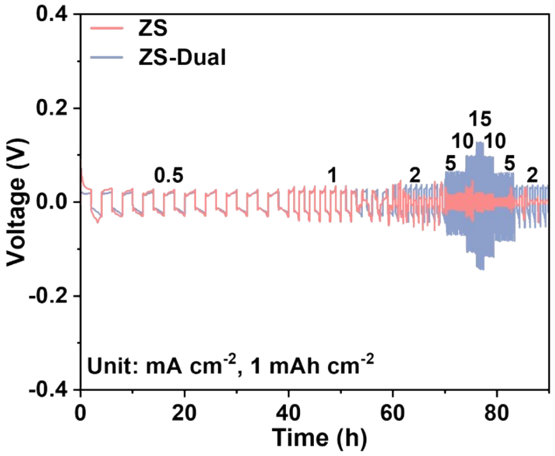

Fig. S38. Rate capability of Zn||Zn cells with 1 mAh cm^{-2}.

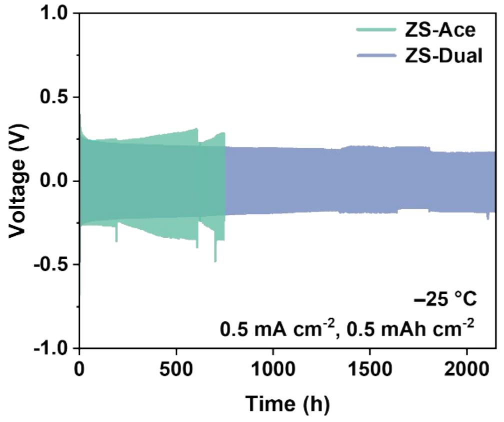

Fig. S39. Galvanostatic Zn plating/stripping in a Zn||Zn symmetric cell at 0.5 mA cm^{-2}, 0.5 mAh cm^{-2} under −25°C in different electrolyte.

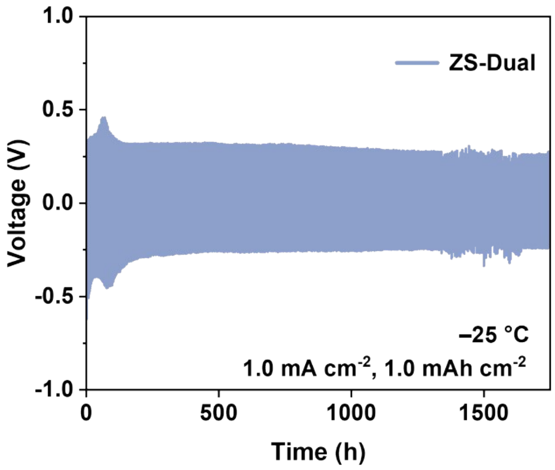

Fig. S40. Galvanostatic Zn plating/stripping in a Zn||Zn symmetric cell at 1 mA cm^{-2}, 1 mAh cm^{-2} under −25°C in ZS-Dual electrolyte.

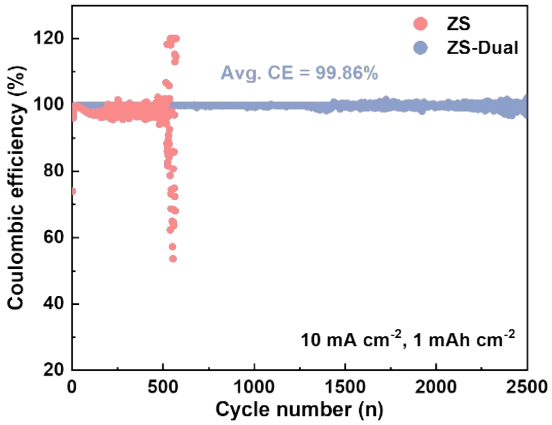

Fig. S41. Zn plating/stripping Coulombic efficiency (CE) in a Zn||Cu asymmetric cell at 10 mA cm^{-2}, and 1 mAh cm^{-2} in ZS and ZS-Dual electrolytes.

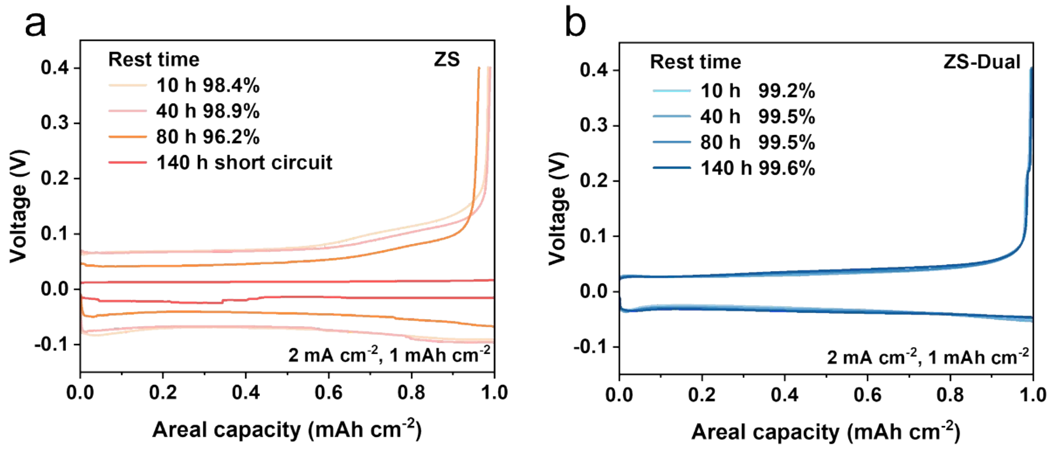

Fig. S42. Galvanostatic voltage profiles in a Zn||Cu asymmetric cell after different rest times in (a) ZS, and (b) ZS-Dual electrolytes.

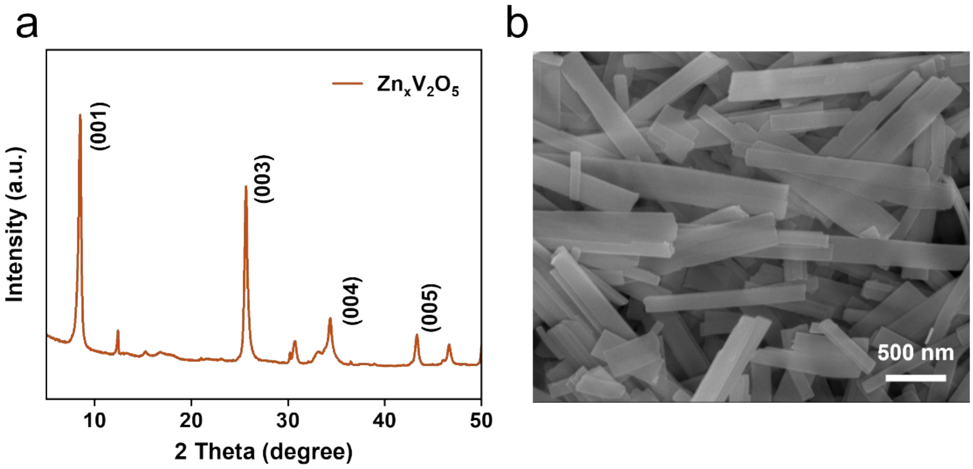

Fig. S43. (a) XRD patterns, and (b) SEM image of ZVO.

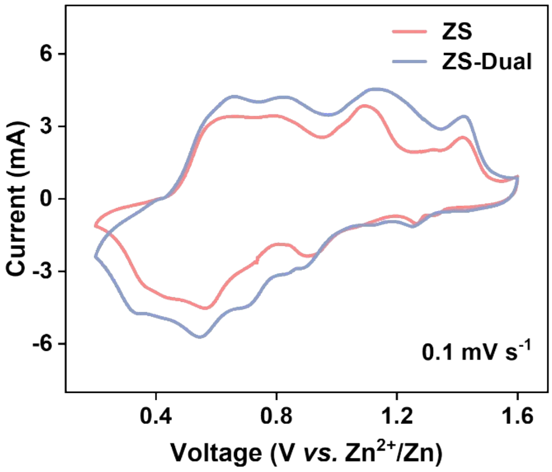

Fig. S44. CV curves of Zn||ZVO full cells in different electrolytes at 0.1 mV s^{-1}.

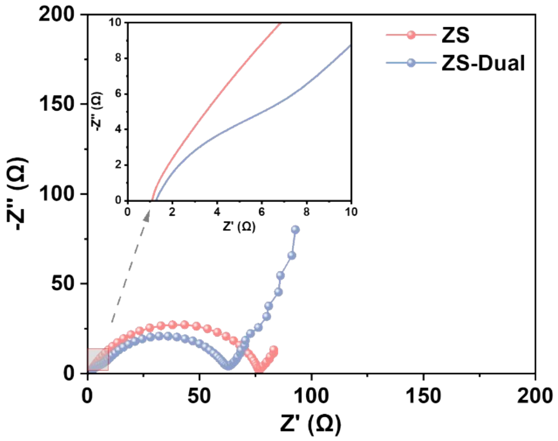

Fig. S45. EIS tests of the Zn||ZVO full cells in ZS and ZS-Dual electrolytes.

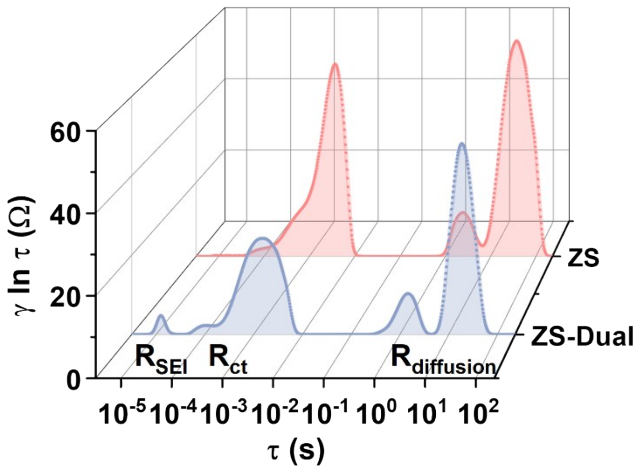

Fig. S46. DRT analysis of full cells in ZS and ZS-Dual electrolytes.

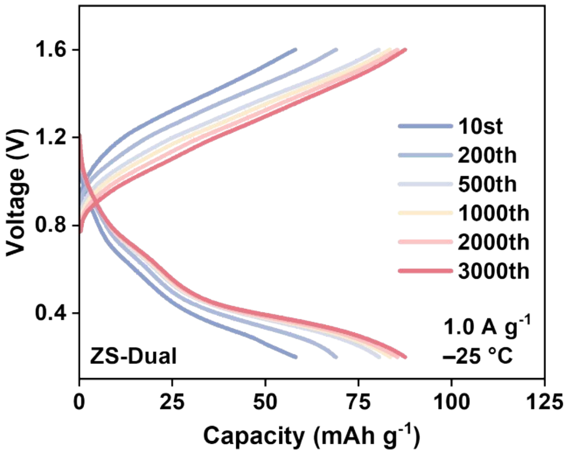

Fig. S47. Charge and discharge voltage profiles of Zn||ZVO full cells at −25 °C with 4.0 A g^{-1}.

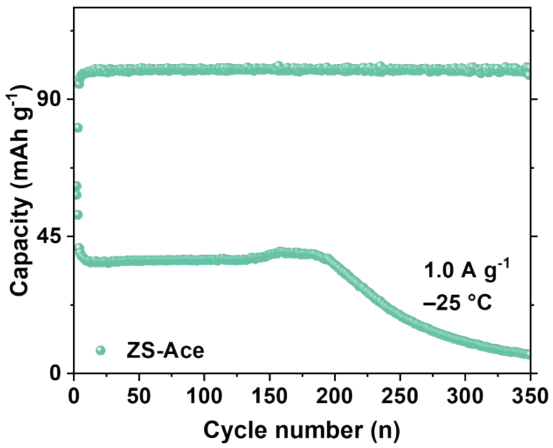

Fig. S48. Cycling performance and corresponding CE of Zn||ZVO full cells in ZS-Ace electrolyte at −25 °C with 0.1 A g^{-1}.

Low temperature conditions moderate the side reactions, but exacerbate the challenges of ion transport kinetics.[16, 17] As a result, the capacity of the Asn-deficient ZS-Ace electrolyte rapidly decays to failure after as few as 200 cycles.

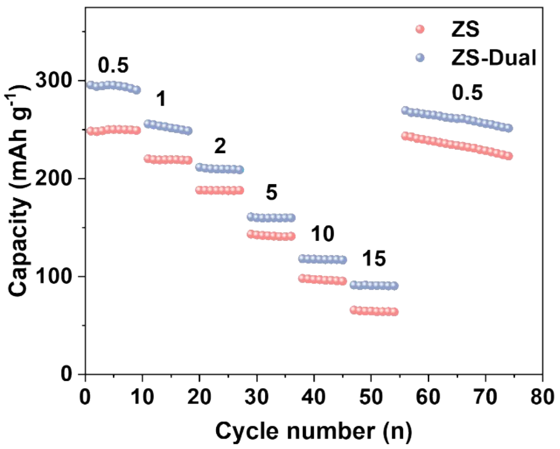

Fig. S49. Rate performance of Zn||ZVO full cells at different current densities.

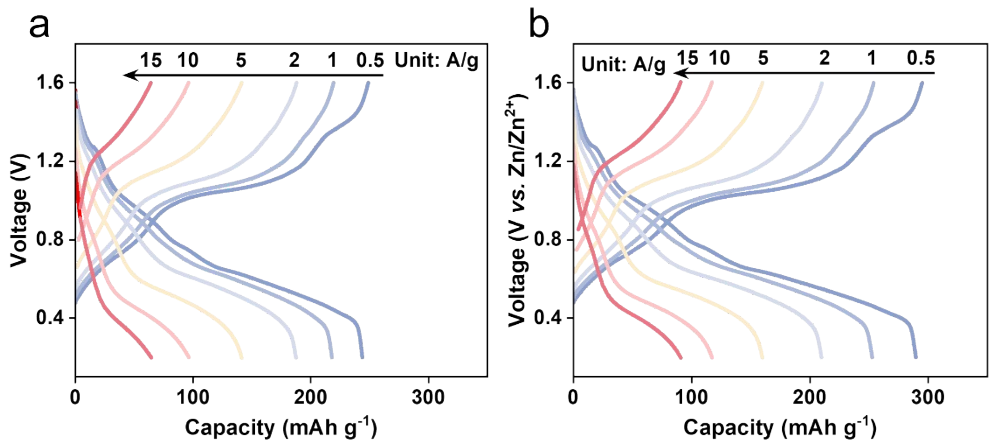

Fig. S50. Corresponding discharge and charge curves at different current densities in (a) ZS and (b) ZS-Dual electrolyte.

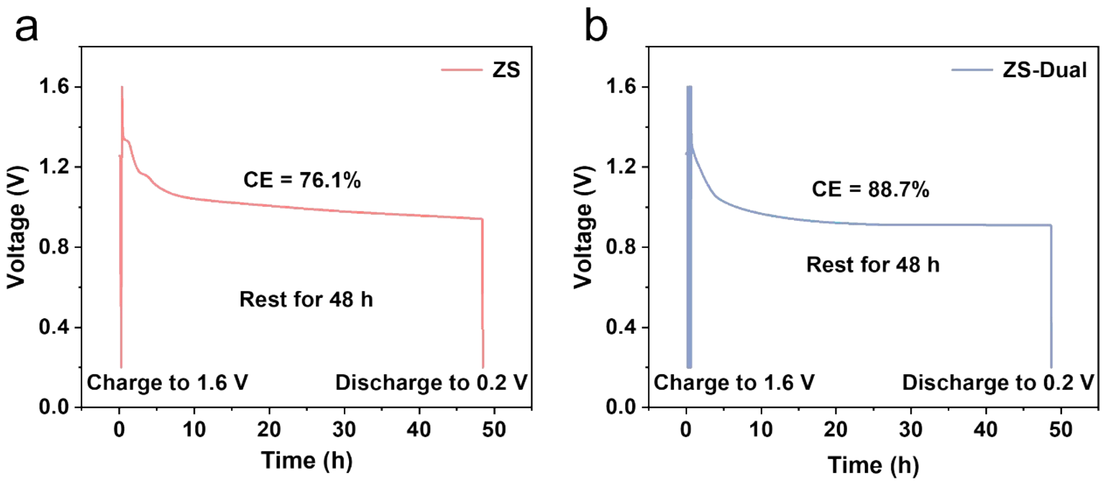

Fig. S51. Self-discharge curves of full cells in the (a) ZS and (b) ZS-Dual electrolyte after 48-h rest.

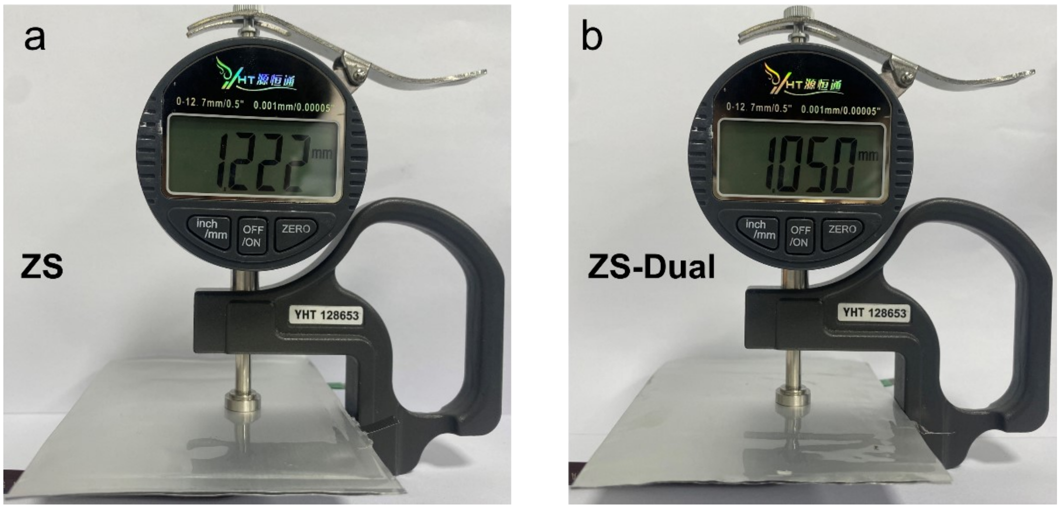

Fig. S52. Digital photos of thickness measurements for pouch cells after cycling in the (a) ZS and (b) ZS-Dual electrolyte.

Table S1. Comparison of Zn plating/stripping performances for this work with Zn-based symmetric cells reported recently.

Electrolyte	Current density (mA cm^{-2})	Area capacity (mAh cm^{-2})	Cycle life (h)	Ref.
0.5 M $Zn(HBS)_2$	5	1	1500	[18]
2 M $Zn(OTF)_2$ in H_2O-ACN (Mass ratio 9:1)	1	1	1200	[19]
2 M $ZnSO_4$ + 10 mM TAA	1	1	1200	[20]
2 M $ZnSO_4$ + 1 mM $NH_3 \cdot H_2O$	1	1	1500	[21]
3 M $ZnSO_4$ + 10 mM α-CD	5	5	200	[22]
1.82 g $Zn(OTF)_2$ in 5 ml H_2O-MeOH (Molar ratio 44:56)	5	5	1000	[23]
1 M $Zn(OTF)_2$ in H_2O-ACE (Volume ratio 1:1)	1	1	800	[24]
2 M $ZnSO_4$ + 0.02 m DMA	1	1	3200	[25]
2 M $ZnSO_4$ + 0.01 M $BMIm^+$	2	1	3000	[26]
1 M $ZnSO_4$ + 20 mM MPS	1	1	4500	[27]
100 ml 2 M $ZnSO_4$ + 90 mg β-CD	4	2	1000	[28]
2 M $ZnSO_4$ + 0.01 M DLP	1	1	1600	[29]
1 M $Zn(OTF)_2$ + 50 mg ml^{-1} Dextran	1	1	850	[30]
2 M $ZnSO_4$ in H_2O-ACE (Volume ratio 97:3)	5	5	500	[31]
1 M $ZnSO_4$ + 60 mM TPPS	1	1	2300	[32]
1 M $ZnSO_4$ + 20 mM Na_3MGDA	0.5	0.5	4080	[33]
1 M $Zn(OTF)_2$ in H_2O-ACE-CPL (Volume ratio 1:1:2)	1	1	2000	[34]
2 M $ZnSO_4$ + 50 mM Hex	1	1	1700	[35]
2 M $ZnSO_4$ + 10 mM DMI	2	1	1600	[36]
1 M $Zn(OTF)_2$ + 1 M $ZnSO_4$ in H_2O-TEP-DMSO (Volume ratio 1:1:1)	5	1	800	[37]

**2 M $ZnSO_4$ + 9 M Ace +0.2 M Asn**	**0.5**	**0.5**	**2150**	**This work**
	**5**	**1**	**2700**	

Table S2. Comparison of electrochemical performance for this work with recently reported Zn-based aqueous batteries.

Electrolyte	Electrode	Capacity mAh g^{-1}	Capacity retention	Ref.
4 M $Zn(OTF)_2$ + 0.25 m TMU	Zn||V_2O_5	207	37% after 2000 cycles at 1 A g^{-1}	38
2 M $ZnSO_4$ in H_2O-PG (Volume ratio 1:1)	Zn||MnO_2	160	85% after 300 cycles at 1 A g^{-1}	39
2 M $ZnSO_4$ + 1 mM Z10	Zn||V_2O_5	264	76% after 1000 cycles at 5 A g^{-1}	40
2 M $ZnSO_4$ + 0.5 M DMI	Zn||VS_2	107	95% after 600 cycles at 1 A g^{-1}	41
3 M $ZnSO_4$ + 0.05 M $Y_2(SO_4)_3$	Zn||$NH_4V_4O_{10}$	277	90% after 2000 cycles at 5 A g^{-1}	42
2 M $ZnSO_4$ + 0.5 mass ratio GA	Zn||V_2O_5	124	65% after 800 cycles at 1 A g^{-1}	43
1 M $ZnSO_4$ + 0.2 M CTAB	Zn||MnO_2	242	100% after 1000 cycles at 1 A g^{-1}	44
1 M $ZnSO_4$ + 25 mM NHP	Zn||MnO_2	115	87% after 1000 cycles at 1 A g^{-1}	45
2 M $ZnSO_4$ + 0.2 M NMI	Zn||MnO_2-PANI	234	100% after 200 cycles at 1 A g^{-1}	46
1 M $ZnSO_4$ + 5 mM THL	Zn||$Zn_xV_2O_5$	250	72% after 2000 cycles at 0.5 A g^{-1}	47
2 M $Zn(OTF)_2$ + 60mM β-CD	Zn||$Zn_xV_2O_5$	141	90% after 850 cycles at 5 A g^{-1}	48
1 m $ZnSO_4$ + 0.1 m HTFSI	Zn||ZnV_6O_9	237	77% after 2000 cycles at 1 A g^{-1}	49
2 M $ZnSO_4$ + 0.2 M $MnSO_4$	Zn||MnO_2	260	90% after 5000 cycles at	50

+ 0.05 M SHP			5 A g^{-1}	
**2 M $ZnSO_4$ + 9 M Ace +0.2 M Asn**	**Zn||$Zn_xV_2O_5$**	**185**	**95% after 4600 cycles at 3 A g^{-1}**	**This work**

Notes and references

1. T. H. Wan, M. Saccoccio, C. Chen and F. Ciucci, Influence of the Discretization Methods on the Distribution of Relaxation Times Deconvolution: Implementing Radial Basis Functions with DRTtools, *Electrochim. Acta*, 2015, **184**, 483-499.
2. G. Kresse and J. Hafner, Ab initio molecular dynamics for open-shell transition metals, *Phys. Rev. B. Condens. Matter.*, 1993, **48**, 13115-13118.
3. P. Hohenberg and W. Kohn, Inhomogeneous Electron Gas, *Phys. Rev.*, 1964, **136**, B864-B871.
4. W. Kohn and L. J. Sham, Self-Consistent Equations Including Exchange and Correlation Effects, *Phys. Rev.*, 1965, **140**, A1133-A1138.
5. P. E. Blochl, Projector augmented-wave method, *Phys. Rev. B. Condens. Matter.*, 1994, **50**, 17953-17979.
6. J. P. Perdew, K. Burke and M. Ernzerhof, Generalized Gradient Approximation Made Simple, *Phys. Rev. Lett.*, 1996, **77**, 3865-3868.
7. A. P. Thompson, H. M. Aktulga, R. Berger, D. S. Bolintineanu, W. M. Brown, P. S. Crozier, P. J. in 't Veld, A. Kohlmeyer, S. G. Moore, T. D. Nguyen, R. Shan, M. J. Stevens, J. Tranchida, C. Trott and S. J. Plimpton, LAMMPS - a flexible simulation tool for particle-based materials modeling at the atomic, meso, and continuum scales, *Comput. Phys. Commun.*, 2022, **271**, 108171.
8. C. S. Babu and C. Lim, Empirical Force Fields for Biologically Active Divalent Metal Cations in Water, *The J. Phys. Chem.*, 2006, **110**, 691-699.
9. W. R. Cannon, B. M. Pettitt and J. A. McCammon, Sulfate Anion in Water: Model Structural, Thermodynamic, and Dynamic Properties, *J. Phys. Chem.*, 1994, **98**, 6225-6230.
10. W. L. Jorgensen and J. Tirado-Rives, Potential energy functions for atomic-level simulations of water and organic and biomolecular systems, *Proc. Natl. Acad. Sci.*, 2005, **102**, 6665-6670.
11. A. I. Jewett, D. Stelter, J. Lambert, S. M. Saladi, O. M. Roscioni, M. Ricci, L. Autin, M. Maritan, S. M. Bashusqeh, T. Keyes, R. T. Dame, J.-E. Shea, G. J. Jensen and D. S. Goodsell, Moltemplate: A Tool for Coarse-Grained Modeling of Complex Biological Matter and Soft Condensed Matter Physics, *J. Mol. Biol.*, 2021, **433**, 166841.
12. L. Martínez, R. Andrade, E. G. Birgin and J. M. Martínez, PACKMOL: A package for building initial configurations for molecular dynamics simulations, *J. Comput. Chem.*, 2009, **30**, 2157-2164.
13. L. J. V. Ahrens-Iwers, M. Janssen, S. R. Tee and R. H. Meißner, ELECTRODE: An electrochemistry package for atomistic simulations, *J. Phys. Chem.*, 2022, **157**, 084801.
14. M. Nezakat, H. Akhiani, S. M. Sabet and J. Szpunar, Electron backscatter and X-ray diffraction studies on the deformation and annealing textures of austenitic stainless steel 310S, *Mater. Charact.*, 2017, **123**, 115-127.
15. M. Zhou, S. Guo, J. Li, X. Luo, Z. Liu, T. Zhang, X. Cao, M. Long, B. Lu, A. Pan, G. Fang, J. Zhou and S. Liang, Surface-Preferred Crystal Plane for a Stable and Reversible Zinc Anode, *Adv. Mater.*, 2021, **33**, 2100187.
16. R. Hou, S. Guo and H. Zhou, Atomic Insights into Advances and Issues in Low-Temperature Electrolytes, *Adv. Energy Mater.*, 2023, **13**, 2300053.
17. M. Li, X. Wang, J. Meng, C. Zuo, B. Wu, C. Li, W. Sun and L. Mai, Comprehensive Understandings of Hydrogen Bond Chemistry in Aqueous Batteries, *Adv. Mater.*, 2024, **36**, 2308628.
18. X. Wang, W. Zhou, L. Wang, Y. Zhang, S. Li, X. Li, Z. Zhao, T. Zhang, H. Jin, X. Song, P. Liang, B. Zhang, D. Zhao and D. Chao, Benchmarking Corrosion with Anionic Polarity Index for Stable and Fast Aqueous Batteries Even in Low-Concentration Electrolyte, *Adv. Mater.*, 2025, **37**, 2501049.
19. J. Zheng, B. Zhang, X. Chen, W. Hao, J. Yao, J. Li, Y. Gan, X. Wang, X. Liu, Z. Wu, Y. Liu, L. Lv, L. Tao, P. Liang, X. Ji, H. Wang and H. Wan, Critical Solvation Structures Arrested Active Molecules for Reversible Zn Electrochemistry, *Nano-Micro Lett.*, 2024, **16**, 145.
20. K. Ren, M. Li, Q. Wang, B. Liu, C. Sun, B. Yuan, C. Lai, L. Jiao and C. Wang, Thioacetamide Additive

Homogenizing Zn Deposition Revealed by In Situ Digital Holography for Advanced Zn Ion Batteries, *Nano-Micro Lett.*, 2024, **16**, 117.

21. R. Chen, W. Zhang, Q. Huang, C. Guan, W. Zong, Y. Dai, Z. Du, Z. Zhang, J. Li, F. Guo, X. Gao, H. Dong, J. Zhu, X. Wang and G. He, Trace Amounts of Triple-Functional Additives Enable Reversible Aqueous Zinc-Ion Batteries from a Comprehensive Perspective, *Nano-Micro Lett.*, 2023, **15**, 81.
22. K. Zhao, G. Fan, J. Liu, F. Liu, J. Li, X. Zhou, Y. Ni, M. Yu, Y.-M. Zhang, H. Su, Q. Liu and F. Cheng, Boosting the Kinetics and Stability of Zn Anodes in Aqueous Electrolytes with Supramolecular Cyclodextrin Additives, *J. Am. Chem. Soc.*, 2022, **144**, 11129-11137.
23. W. Xu, J. Li, X. Liao, L. Zhang, X. Zhang, C. Liu, K. Amine, K. Zhao and J. Lu, Fluoride-Rich, Organic–Inorganic Gradient Interphase Enabled by Sacrificial Solvation Shells for Reversible Zinc Metal Batteries, *J. Am. Chem. Soc.*, 2023, **145**, 22456-22465.
24. X. Cao, W. Xu, D. Zheng, F. Wang, Y. Wang, X. Shi and X. Lu, Weak Solvation Effect Induced Optimal Interfacial Chemistry Enables Highly Durable Zn Anodes for Aqueous Zn-Ion Batteries, *Angew. Chem. Int. Ed.*, 2024, **63**, e202317302.
25. T. Wei, H. Zhang, Y. Ren, L. e. Mo, Y. He, P. Tan, Y. Huang, Z. Li, D. Zhu and L. Hu, Building Near-Unity Stacked (002) Texture for High-Stable Zinc Anode, *Adv. Funct. Mater.*, 2024, **34**, 2312506.
26. H. Zhang, Y. Zhong, J. Li, Y. Liao, J. Zeng, Y. Shen, L. Yuan, Z. Li and Y. Huang, Inducing the Preferential Growth of Zn (002) Plane for Long Cycle Aqueous Zn-Ion Batteries, *Adv. Energy Mater.*, 2023, **13**, 2203254.
27. Y. Lin, Y. Li, Z. Mai, G. Yang and C. Wang, Interfacial Regulation via Anionic Surfactant Electrolyte Additive Promotes Stable (002)-Textured Zinc Anodes at High Depth of Discharge, *Adv. Energy Mater.*, 2023, **13**, 2301999.
28. C. Meng, W. He, L. Jiang, Y. Huang, J. Zhang, H. Liu and J.-J. Wang, Ultra-Stable Aqueous Zinc Batteries Enabled by β-Cyclodextrin: Preferred Zinc Deposition and Suppressed Parasitic Reactions, *Adv. Funct. Mater.*, 2022, **32**, 2207732.
29. Q. Zhu, G. Sun, S. Qiao, D. Wang, Z. Cui, W. Zhang and J. Liu, Selective Shielding of the (002) Plane Enabling Vertically Oriented Zinc Plating for Dendrite-Free Zinc Anode, *Adv. Mater.*, 2024, **36**, 2308577.
30. J. Li, Z. Guo, J. Wu, Z. Zheng, Z. Yu, F. She, L. Lai, H. Li, Y. Chen and L. Wei, Dextran: A Multifunctional and Universal Electrolyte Additive for Aqueous Zn Ion Batteries, *Adv. Energy Mater.*, 2023, **13**, 2301743.
31. H. Cao, X. Zhang, B. Xie, X. Huang, F. Xie, Y. Huo, Q. Zheng, R. Zhao, Q. Hu, L. Kang, S. Liu and D. Lin, Unraveling the Solvation Structure and Electrolyte Interface through Carbonyl Chemistry for Durable and Dendrite-Free Zn Anode, *Adv. Funct. Mater.*, 2023, **33**, 2305683.
32. X. Zhao, Y. Wang, C. Huang, Y. Gao, M. Huang, Y. Ding, X. Wang, Z. Si, D. Zhou and F. Kang, Tetraphenylporphyrin-based Chelating Ligand Additive as a Molecular Sieving Interfacial Barrier toward Durable Aqueous Zinc Metal Batteries, *Angew. Chem. Int. Ed.*, 2023, **62**, e202312193.
33. H. Liang, J. Wu, J. Xu, J. Li, J. Wang, J. Cai, Y. Long, X. Yu and Z. Yang, Inert Group-Containing Electrolyte Additive Enabling Stable Aqueous Zinc-Ion Batteries, *Small*, 2024, **20**, 2307322.
34. S. Wang, G. Liu, W. Wan, X. Li, J. Li and C. Wang, Acetamide-Caprolactam Deep Eutectic Solvent-Based Electrolyte for Stable Zn-Metal Batteries, *Adv. Mater.*, 2024, **36**, 2306546.
35. Q. Hu, J. Hu, L. Li, Q. Ran, Y. Ji, X. Liu, J. Zhao and B. Xu, In-depth study on the regulation of electrode interface and solvation structure by hydroxyl chemistry, *Energy Storage Mater.*, 2023, **54**, 374-381.
36. Y. Meng, M. Wang, J. Wang, X. Huang, X. Zhou, M. Sajid, Z. Xie, R. Luo, Z. Zhu, Z. Zhang, N. A. Khan, Y. Wang, Z. Li and W. Chen, Robust bilayer solid electrolyte interphase for Zn electrode with high utilization and efficiency, *Nat. Commun.*, 2024, **15**, 8431.
37. L. Lin, Z. Shao, S. Liu, P. Yang, K. Zhu, W. Zhuang, C. Li, G. Guo, W. Wang, G. Hong, B. Wu, Q. Zhang and Y. Yao,

High-Entropy Aqueous Electrolyte Induced Formation of Water-Poor Zn2+ Solvation Structures and Gradient Solid-Electrolyte Interphase for Long-Life Zn-Metal Anodes, *Angew. Chem. Int. Ed.*, 2025, **64**, e202425008.

38. J. Yang, Y. Zhang, Z. Li, X. Xu, X. Su, J. Lai, Y. Liu, K. Ding, L. Chen, Y.-P. Cai and Q. Zheng, Three Birds with One Stone: Tetramethylurea as Electrolyte Additive for Highly Reversible Zn-Metal Anode, *Adv. Funct. Mater.*, 2022, **32**, 2209642.
39. J. Li, S. Zhou, Y. Chen, X. Meng, A. Azizi, Q. He, H. Li, L. Chen, C. Han and A. Pan, Self-Smoothing Deposition Behavior Enabled by Beneficial Potential Compensating for Highly Reversible Zn-Metal Anodes, *Adv. Funct. Mater.*, 2023, **33**, 2307201.
40. L. Tao, K. Guan, R. Yang, Z. Guo, L. Wang, L. Xu, H. Wan, J. Zhang, H. Wang, L. Hu, P. J. Dyson, M. K. Nazeeruddin and H. Wang, Dual-protected zinc anodes for long-life aqueous zinc ion battery with bifunctional interface constructed by zwitterionic surfactants, *Energy Storage Mater.* 2023, **63**, 102981.
41. K. Lu, C. Chen, Y. Wu, C. Liu, J. Song, H. Jing, P. Zhao, B. Liu, M. Xia, Q. Hao and W. Lei, Versatile 1, 3-dimethyl-2-imidazolidinone electrolyte additive: Enables extremely long life zinc metal batteries with different substrates, *Chem. Eng. J.*, 2023, **457**, 141287.
42. Y. Ding, X. Zhang, T. Wang, B. Lu, Z. Zeng, Y. Tang, J. Zhou and S. Liang, A dynamic electrostatic shielding layer toward highly reversible Zn metal anode, *Energy Storage Mater.,* 2023, **62**, 102949.
43. H. Zheng, Y. Huang, J. Xiao, W. Zeng, X. Li, X. Li, M. Wang and Y. Lin, Multi-protection of zinc anode via employing a natural additive in aqueous zinc ion batteries, *Chem. Eng. J.*, 2023, **468**, 143834.
44. Z. Liu, R. Wang, Y. Gao, S. Zhang, J. Wan, J. Mao, L. Zhang, H. Li, J. Hao, G. Li, L. Zhang and C. Zhang, Low-Cost Multi-Function Electrolyte Additive Enabling Highly Stable Interfacial Chemical Environment for Highly Reversible Aqueous Zinc Ion Batteries, *Adv. Funct. Mater.*, 2023, **33**, 2308463.
45. W. Zhang, Y. Dai, R. Chen, Z. Xu, J. Li, W. Zong, H. Li, Z. Li, Z. Zhang, J. Zhu, F. Guo, X. Gao, Z. Du, J. Chen, T. Wang, G. He and I. P. Parkin, Highly Reversible Zinc Metal Anode in a Dilute Aqueous Electrolyte Enabled by a pH Buffer Additive, *Angew. Chem. Int. Ed.*, 2023, **62**, e202212695.
46. M. Zhang, H. Hua, P. Dai, Z. He, L. Han, P. Tang, J. Yang, P. Lin, Y. Zhang, D. Zhan, J. Chen, Y. Qiao, C. C. Li, J. Zhao and Y. Yang, Dynamically Interfacial pH-Buffering Effect Enabled by N-Methylimidazole Molecules as Spontaneous Proton Pumps toward Highly Reversible Zinc-Metal Anodes, *Adv. Mater.*, 2023, **35**, 2208630.
47. H. Li, Y. Ren, Y. Zhu, J. Tian, X. Sun, C. Sheng, P. He, S. Guo and H. Zhou, A Bio-Inspired Trehalose Additive for Reversible Zinc Anodes with Improved Stability and Kinetics, *Angew. Chem. Int. Ed.*, 2023, **62**, e202310143.
48. J. Luo, L. Xu, Y. Yang, S. Huang, Y. Zhou, Y. Shao, T. Wang, J. Tian, S. Guo, J. Zhao, X. Zhao, T. Cheng, Y. Shao and J. Zhang, Stable zinc anode solid electrolyte interphase via inner Helmholtz plane engineering, *Nat. Commun.*, 2024, **15**, 6471.
49. Q. Nian, X. Luo, D. Ruan, Y. Li, B.-Q. Xiong, Z. Cui, Z. Wang, Q. Dong, J. Fan, J. Jiang, J. Ma, Z. Ma, D. Wang and X. Ren, Highly reversible zinc metal anode enabled by strong Brønsted acid and hydrophobic interfacial chemistry, *Nat. Commun.*, 2024, **15**, 4303.
50. M. Chen, M. Yang, X. Han, J. Chen, P. Zhang and C.-P. Wong, Suppressing Rampant and Vertical Deposition of Cathode Intermediate Product via PH Regulation Toward Large-Capacity and High-Durability Zn//MnO2 Batteries, *Adv. Mater.*, 2024, **36**, 2304997.
